# Multimodal transcriptomics reveal neurogenic aging trajectories and age-related regional inflammation in the dentate gyrus

**DOI:** 10.1038/s41593-024-01848-4

**Published:** 2025-01-06

**Authors:** Yicheng Wu, Vladyslav I. Korobeynyk, Margherita Zamboni, Felix Waern, John Darby Cole, Sarah Mundt, Melanie Greter, Jonas Frisén, Enric Llorens-Bobadilla, Sebastian Jessberger

**Affiliations:** 1 Laboratory of Neural Plasticity, Faculties of Medicine and Science, Brain Research Institute, University of Zurich, Zurich, Switzerland; 2 Department of Cell and Molecular Biology, Karolinska Institute, Stockholm, Sweden; 3 Institute of Experimental Immunology, University of Zurich, Zurich, Switzerland

**Keywords:** Neural ageing, Neural stem cells

## Abstract

The mammalian dentate gyrus (DG) is involved in certain forms of learning and memory, and DG dysfunction has been implicated in age-related diseases. Although neurogenic potential is maintained throughout life in the DG as neural stem cells (NSCs) continue to generate new neurons, neurogenesis decreases with advancing age, with implications for age-related cognitive decline and disease. In this study, we used single-cell RNA sequencing to characterize transcriptomic signatures of neurogenic cells and their surrounding DG niche, identifying molecular changes associated with neurogenic aging from the activation of quiescent NSCs to the maturation of fate-committed progeny. By integrating spatial transcriptomics data, we identified the regional invasion of inflammatory cells into the hippocampus with age and show here that early-onset neuroinflammation decreases neurogenic activity. Our data reveal the lifelong molecular dynamics of NSCs and their surrounding neurogenic DG niche with age and provide a powerful resource to understand age-related molecular alterations in the aging hippocampus.

## Abstract (web summary)

Multimodal transcriptomics unveil the molecular dynamics of neural stem cells and their surrounding niche in the aging mouse hippocampus and provide a resource to understand age-related molecular changes.

## Main

The hippocampus is a key brain structure underlying the encoding of declarative memories, such as biographical events and semantic facts, and is required for spatial learning^1–6^. The hippocampus also plays a pivotal role in mood control^7–9^. The dentate gyrus (DG), which is the hippocampal subregion that receives the main inputs from cortical association areas, is one of the few regions in the adult mammalian brain that harbors neurogenic neural stem cells (NSCs)^10,11^. NSCs produce new neurons throughout life that integrate into pre-existing neural networks, providing structural and functional plasticity to hippocampal circuits. Aging has been associated with reduced levels of neurogenesis and a decline in hippocampus-dependent learning and memory^12–14^. Moreover, several age-related diseases, including Alzheimer’s disease and major depression, have been linked to impaired plasticity of the aging hippocampus^11,15–19^. However, the molecular alterations that occur from early adulthood into old age within the DG niche, required to sustain lifelong neurogenesis and to ensure proper circuit function, remain poorly understood.

Recent progress in single-cell RNA sequencing (scRNA-seq) enabled genome-wide transcriptome analyses of individual cells and proved to be a powerful tool in mice, monkeys and humans to identify developmental trajectories of the hippocampus and neurogenic cells within the DG^20–26^. Indeed, scRNA-seq was used in the other main neurogenic niche of rodents—the subventricular zone (SVZ) lining the lateral ventricles—to characterize age-related changes in gene expression and to identify novel regulators of neurogenesis in the context of aging^27,28^. Despite its usefulness to identify molecular signatures of individual cells, current scRNA-seq-based approaches require the destruction of tissues to isolate single cells or nuclei and, therefore, lack spatial information about the localization of sequenced cells within the analyzed brain area. To overcome this limitation, several approaches have been established that allow for genome-wide spatially resolved transcriptomics or multiplexed in situ analyses of genes and/or proteins^29,30^. Spatially resolved transcriptomics have been used to study cellular interactions and regional differences of tissues in health and disease^31,32^. Furthermore, multiplexed, candidate-based approaches to analyze the expression of multiple proteins in individual cells have been used to identify regional and single-cell heterogeneity of age-related changes in the aging DG^33^. However, genome-wide data with sufficient spatial resolution are currently missing for the mammalian DG across the adult lifespan. In the present study, we used whole-cell scRNA-seq combined with spatially resolved transcriptomics in young adult, middle-aged and aged mice to characterize age-associated molecular changes in the DG. Our data reveal heterogeneity in the course of molecular changes associated with age; identify regional inflammatory cell invasion to be associated with age and to be sufficient to reduce neurogenic activity; and provide a powerful resource to reveal the mechanisms that are linked with aging in the mammalian hippocampus.

## Results

### Transcriptional architecture of the aging DG

**Fig. 1:**
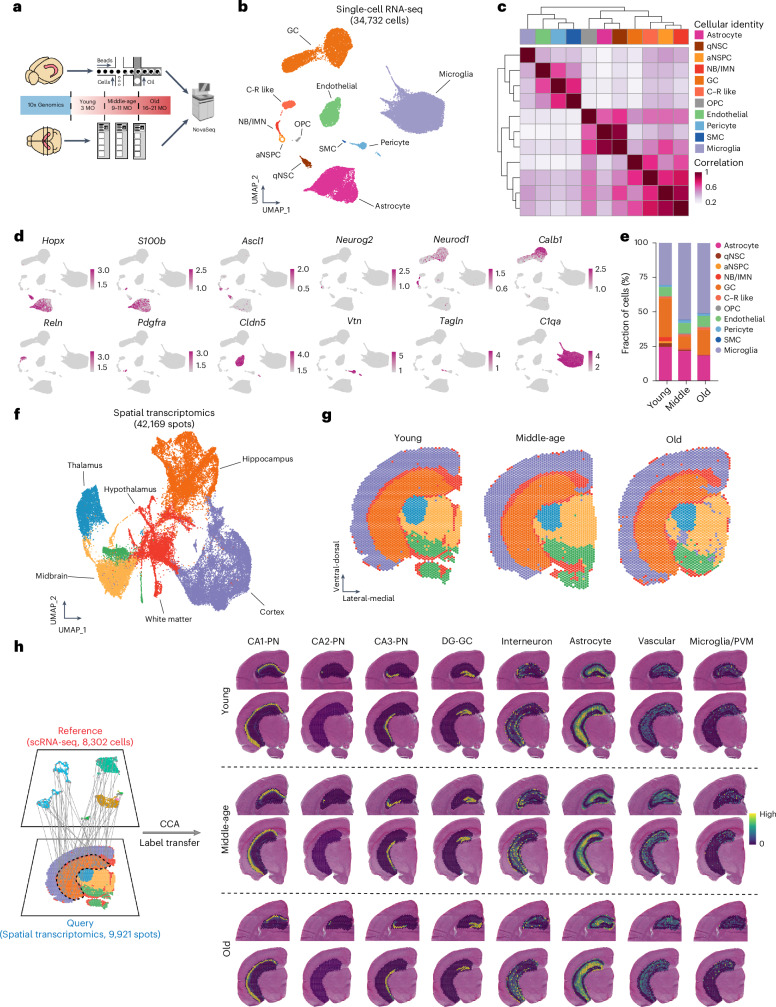
Multimodal transcriptomics of the mouse hippocampal neurogenic niche during aging. **a**, Schematic illustration summarizing experimental design of the multimodal transcriptional atlas of mouse hippocampal neurogenic niche. **b**, UMAP visualization of all DG resident cells in the scRNA-seq dataset. **c**, Hierarchical clustering of all DG resident cells showing distinct transcriptional profiles among all 11 cell types. **d**, Feature plots of selected genes showing cell-type-specific expression profiles of all DG resident cells. Color gradient indicates the log-normalized gene expression level. **e**, Relative proportions of all cell populations in the mouse DG. **f**, UMAP visualization of all spots of the whole mouse brain in the ST dataset. **g**, Spatial projection of all spots into each age. **h**, Spatial mapping of annotated hippocampal cell populations from the Yao et al.^35^ dataset to the current ST dataset using the Seurat CCA tool. Computed enrichment score of each cell type (color gradient) to hippocampus shown over the H&E images. CCA, canonical correlation analysis; C–R, Cajal–Retzius; MO, months old; OPC, oligodendrocyte precursor cell; PN, pyramidal neuron; SMC, smooth muscle cell. Source data

**Extended Data Fig. 1:**
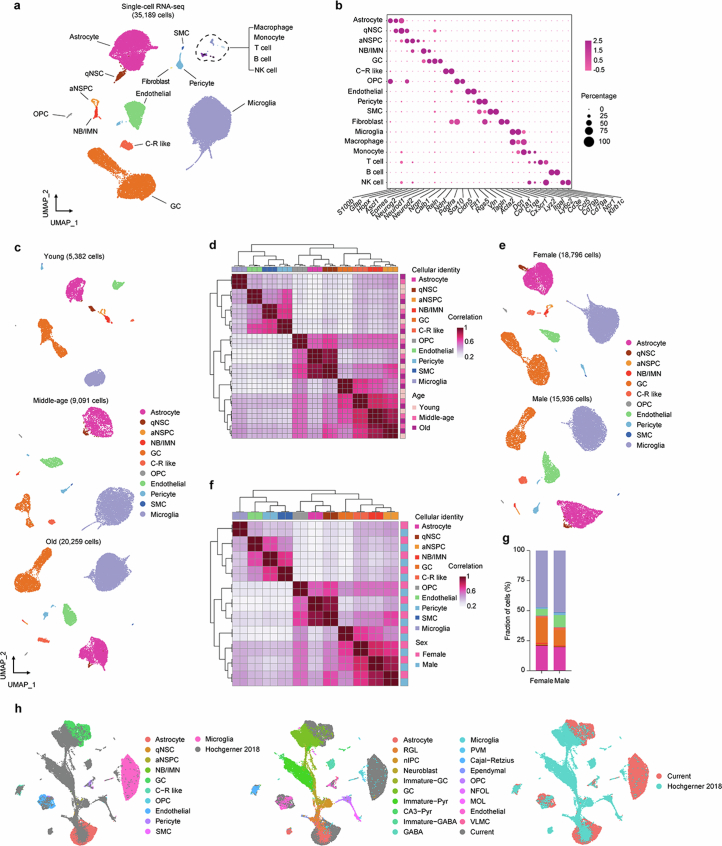
Single-cell transcriptomics of the mouse dentate gyrus. **a**, UMAP visualization of all cells in the scRNA-seq dataset. Periphral immune cell populations are highlighted. **b**, Dot plots of selected genes showing cell type-specific expression patterns. Color gradient indicates the log-normalized gene expression level. **c**, UMAP visualization of all DG resident cell types in individual age. **d**, Pearson correlation of average gene expression profile between different ages. **e**, UMAP visualization of all DG resident cell types in each sex group. **f**, Pearson correlation of average gene expression profile between different sexes. **g**, Relative proportions of all DG resident cell types in each sex group. **h**, UMAP visualization of integration of the current and Hochgerner 2018 dataset. Left: Highlighting cells from the current dataset; Middle: Highlighting cells Hochgerner 2018 dataset; Right: Visualization of dataset origin in the UMAP space. aNSPC, active neural stem and progenitor cells, CA, cornu Ammonis, C-R, Cajal-Retzius, GABA, GABAergic neuron, GC, granule cell, MOL, mature oligodendrocyte, NB/IMN, neuroblast and immature neuron, NFOL, newly formed oligodendrocyte, nIPC, neurogenic intermediate progenitor cell, OPC, oligodendrocyte precursor, PVM, perivascular macrophage, Pyr, pyramidal neuron, qNSC, quiescent neural stem cell, RGL, radial glia-like, scRNA-seq, single-cell RNA-sequencing, SMC, smooth muscle cell, UMAP, uniform manifold approximation and projection and VLMC, vascular and leptomeningeal cell. Source data

We isolated whole cells from young adult (3-month-old (3 MO)), middle-aged (9–11 MO) and old (16–21 MO) C57Bl/6 mice of mixed sex to perform scRNA-seq (Fig. 1a and Supplementary Fig. 1). After filtering out poor-quality cells and doublets, we obtained transcriptomes of 35,189 cells that clustered into 17 distinct cell populations (Extended Data Fig. 1a,b). Of note, some of them were peripheral immune cells that could represent either contamination or infiltration. Thus, we first focused on DG resident cell populations that consisted of 34,732 cells from 11 distinct cell populations (Fig. 1b). These cell populations were well separated by their global transcriptome (Fig. 1c). Top marker genes for each cell type were summarized (Fig. 1d and Supplementary Table 2). Relative proportions of identified cells were dependent on age, with a substantial decrease of cells of the neurogenic lineage—for example, quiescent neural stem cell (qNSC), active neural stem/progenitor cell (aNSPC) and neuroblast/immature neuron (NB/IMN)—as expected (Fig. 1e). Despite the proportional difference, all DG resident populations partitioned into distinct uniform manifold approximation and projection (UMAP) spaces after re-clustering according to individual ages and displayed conserved transcriptional structure (Extended Data Fig. 1c,d). Sex only mildly influenced the relative frequency of sequenced cell populations while the transcriptional structure remained conserved (Extended Data Fig. 1e–g). Proportional changes of distinct cell populations may be used to reflect relative changes in cellular compositions (even though absolute sequenced cell numbers cannot be used to draw conclusions regarding the composition of the tissue given differences in dissociation properties). However, such interpretation may be affected by differential dissociation efficiencies in different tissue and cell types. Thus, we used proportional changes in this study only as the starting point for further tissue validation using immunohistochemistry. To this end, we performed tissue validation of several cell types, including all three neurogenic populations and microglia. These histological results were consistent with scRNA-seq data. Robustness of annotation was further confirmed by high homology with previously obtained perinatal, juvenile and adult hippocampal scRNA-seq data^34^ (Extended Data Fig. 1h).

**Extended Data Fig. 2:**
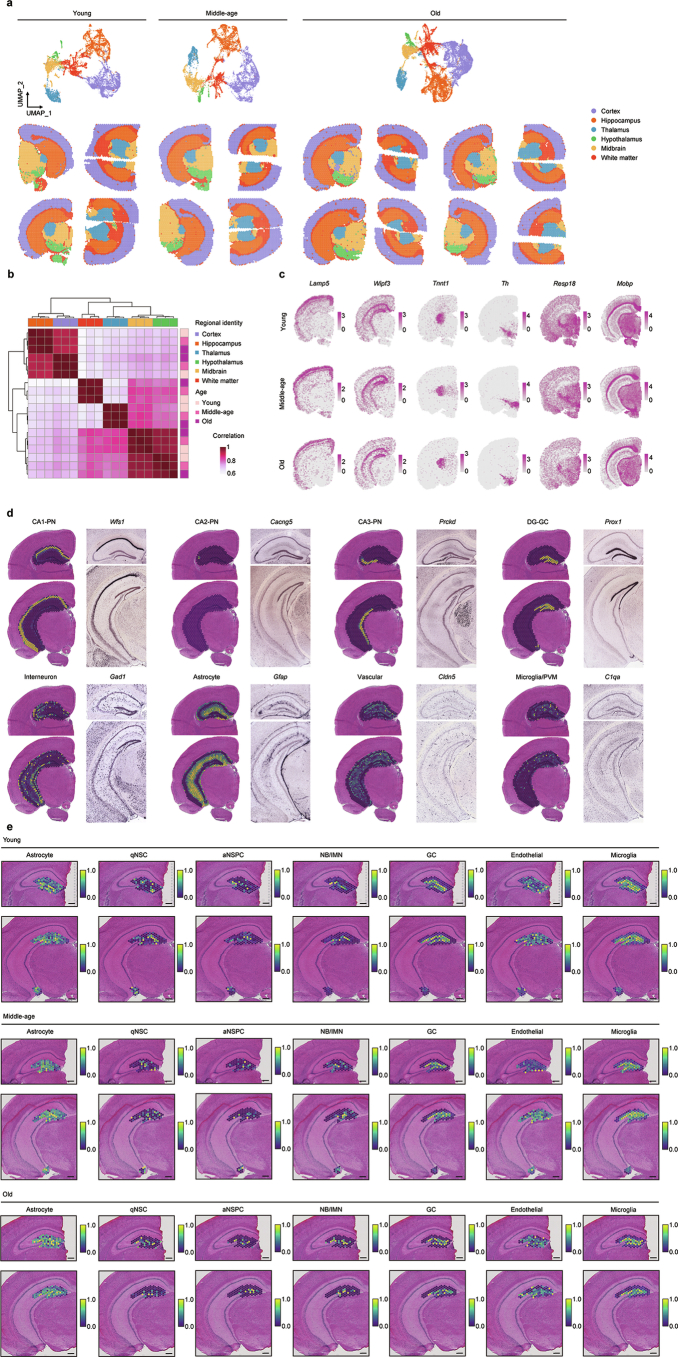
Spatial transcriptomics of the mouse dentate gyrus. **a**, Upper: UMAP visualization of all spots after re-clustering according to individual ages showing concordance of their regional identity across different ages. Lower: Spatial projection of all spots into each Visium section. **b**, Hierarchical clustering of all spots showing distinct transcriptional structure among all 6 regional identities and concordance between different ages. **c**, Featured expression patterns of selected genes representing each of the six identified regions. Color gradient indicates the log-normalized gene expression level. **d**, Validation of selected region-specific genes using Allen Brain Atlas *in situ* hybridization database. **e**, Cell type mapping of the spatial transcriptomics data using tangram. Estimated mapping probability of each cell type (color gradient) to DG shown over the H&E images. aNSPC, active neural stem and progenitor cells, CA, cornu Ammonis, DG, dentate gyrus, GC, granule cell, H&E, Haematoxylin and eosin, NB/IMN, neuroblast and immature neuron, PN, pyramidal neuron and PVM, perivascular macrophage. Scale bars, (**d**) 100 μm and (**e**) 200 μm.

In parallel, we obtained spatial transcriptomes throughout the adult lifespan using cage littermates of the scRNA-seq experiment (Fig. 1a and Supplementary Fig. 2). For all timepoints, 16 slides of 24 coronal sections were acquired, yielding 42,169 spots (Fig. 1f). Similar to scRNA-seq analyses within the DG, we identified six major clusters upon unsupervised clustering that were highly correlated to their anatomical identities by manual annotation (for example, cortex, hippocampus, midbrain, thalamus, hypothalamus and white matter) and displayed featured gene expression patterns conserved across all ages (Fig. 1g and Extended Data Fig. 2a–c). Focusing on the hippocampus, spatial transcriptomes captured cell-type-specific expression patterns of hippocampal subregions and could even distinguish between different highly similar cornu ammonis (CA) regions—for example, CA1 and CA2—in all three ages when aligned with previously obtained data (Fig. 1h and Extended Data Fig. 2d)^35^. We also performed cell type mapping using the Tangram algorithm^36^ to characterize the spatial distribution of dentate cell types within the DG (Extended Data Fig. 2e). Thus, the global transcriptional architecture of the DG and the regional identity of the mouse brain is maintained into old age, allowing for the in-depth analyses of differentially expressed genes (DEGs) with advancing age.

### Neurogenic identities are preserved across the adult lifespan

**Fig. 2:**
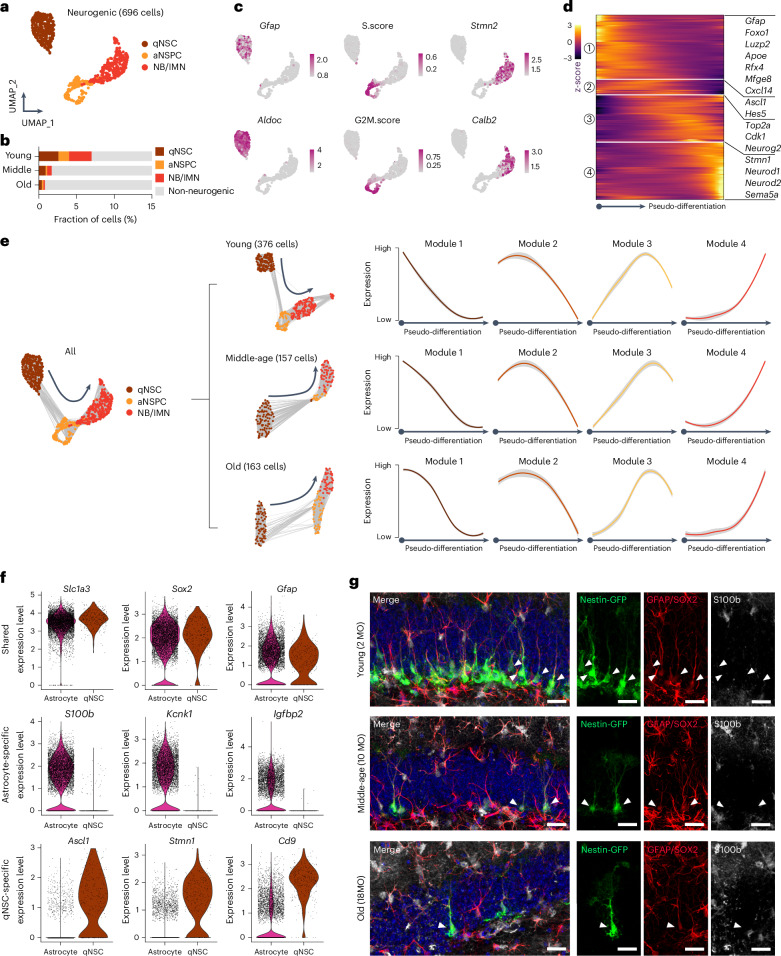
Neurogenic programs are preserved in the aging DG. **a**, UMAP visualization of the whole neurogenic lineages. **b**, Relative proportions of each neurogenic cell type in the mouse DG. **c**, Expression of cell-type-specific genes. Color gradient indicates the log-normalized gene expression level. **d**, Heatmap shows gene expression dynamics of neurogenic lineage progression across pseudotime. Numbers in circle indicate gene modules. Color gradient indicates the z-score of gene expression. **e**, SNN graphs of the whole neurogenic populations (left) and the individual age (right) showing that the uni-directional neurogenic trajectory is preserved in advancing ages as well as the temporal expression patterns of individual pseudotime modules. **f**, Violin plots showing three classes of DEGs between astrocytes and qNSCs. Upper, shared genes include Slc1a3, Sox2 and Gfap; middle, astrocyte-specific genes include S100b, Kcnk1 and Igfbp2; lower, qNSC-specific genes include Ascl1, Stmn1 and Cd9. **g**, Immunofluorescent staining showing that most radial NSCs (GFAP^+^SOX2^+^) labeled by Nestin-GFP in the SGZ across all three ages do not overlap with astrocytic marker S100b (*n* = 3 mice for each condition). Arrowheads indicate the soma of qNSCs. Scale bars, 20 μm. MO, months old. Source data

**Extended Data Fig. 3:**
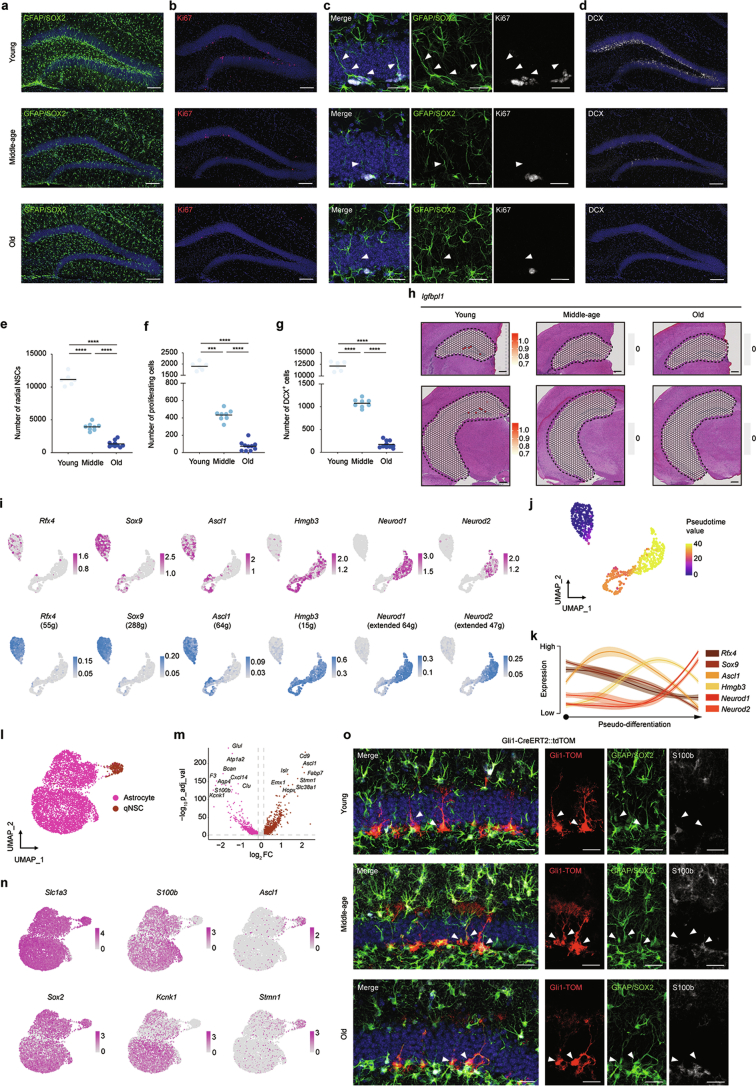
Both neurogenic activity and distinct molecular profile between astrocytes and qNSCs are largely preserved with advancing age. **a-d**, Representative images of neurogenic lineages in SGZ of (upper) young, (middle) middle-age and (lower) old mice showing decreased neurogenesis with aging. (**a**) Radial NSCs were labeled by GFAP and SOX2 with radial morphology in SGZ. Note that GFAP and SOX2 are detected simulataneously (SOX2: nucleus; GFAP: radial process) in the same channel to facilitate identification of radial glia-like progenitors, as used by others before^23^. (**b**) aNSPCs were labeled by Ki67 including (**c**) radial and non-radial proliferating progenitors. Radial and non-radial aNSPCs were distinguished by the existence of the radially oriented GFAP-process (pointed out by arrowhead). (**d**) NB/IMNs were labeled by DCX. **e-g**, Quantification of (**e**) radial NSCs (young: 11151 ± 375 cells; middle-age: 3923 ± 200 cells; old: 1324 ± 154 cells; two-tailed unpaired t-test with Welch’s correction, ****P < 0.0001 between young and middle-age, ****P < 0.0001 between middle-age and old, ****P < 0.0001 between young and old), (**f**) aNSPC (young: 1902 ± 73 cells; middle-age: 433 ± 22 cells; old: 74 ± 17 cells; two-tailed unpaired t-test with Welch’s correction, ****P < 0.0001 between young and middle-age, ****P < 0.0001 between middle-age and old, ****P < 0.0001 between young and old), and (**g**) NB/IMN (young: 12131 ± 392 cells; middle-age: 1079 ± 32 cells; old: 173 ± 25 cells; two-tailed unpaired t-test with Welch’s correction, ****P < 0.0001 between young and middle-age, ****P < 0.0001 between middle-age and old, ****P < 0.0001 between young and old) in SGZ of mice in different ages. **h**, Spatial feature plots of *Igfbpl1* showing age-dependent decrease in DG. Color gradient indicates the log-normalized gene expression level. **i**, Feature plots of selected TFs and their GRNs showing good agreement between individual gene expression and their GRN activity. Color gradient indicates the (upper) log-normalized gene expression level and (lower) GRN activity score. **j**, Projection of pseudotime value in the UMAP space. **k**, Gene expression dynamics along pseudotemporal trajectory of selective genes representing qNSC (Rfx4 and Sox9), aNSPC (Ascl1 and Hmgb3) and NB/IMN (Neurod1 and Neurod2). Shading indicates 95% confidence interval. **l**, UMAP plot of astrocytes and qNSCs showing distinct molecular profiles. **m**, Volcano plot showing DEGs between astrocytes and qNSCs. For all dots shown in color by an adjusted *P* < 0.05 with Bonferroni correction, MAST differential test. **n**, Feature plots of selected genes showing (left) shared, (middle) astrocyte-specific and (right) qNSC-specific genes. Color gradient indicates the log-normalized gene expression level. **o**, Immunofluorescent staining showing the majority of radial NSCs (GFAP^+^SOX2^+^) labeled by Gli1-tdTOM in the SGZ across all three ages do not overlap with astrocytic marker, S100b (n = 3 mice for each condition). DEG, differentially expressed gene, GFP, green fluorescent protein, GRN, gene regulatory network, MO, month-old, qNSC, quiescent neural stem cell, Sac, sacrifice, TOM, tomato, UMAP, uniform manifold approximation and projection, Scale bars, (**a**) 100 μm, (**b**-**d** and **o**) 20 μm and (**h**) 200 μm. Source data

Neurogenic lineages can be resolved into qNSCs, aNSPCs and NB/IMNs (Fig. 2a). Their abundance decreased with advancing age based on relative sequencing frequencies, a finding that was confirmed with histological analyses (Fig. 2b and Extended Data Fig. 3a–g) and spatial transcriptomics (ST) by the measurement of *Igfbpl1*, a neuroblast-specific gene (Extended Data Fig. 3h)^34^. qNSCs can be distinguished from aNSPCs based on the upregulation of astrocytic/stem cell markers (for example, *Gfap* and *Aldoc*) and the lack of cell cycle activity and neuronal specification (for example, *Stmn2* and *Calb2*) (Fig. 2c). Gene regulatory networks (GRNs) of transcription factors (TFs) consisting of TFs, their co-factors and downstream targets were shown to detect subtle molecular variations more accurately than individual genes^37,38^. Indeed, we confirmed our annotation using individual marker genes by their corresponding GRNs (Extended Data Fig. 3i). Next, we constructed the lineage trajectory (pseudo-differentiation) of the entire neurogenic lineage and identified a molecular progression ordered from qNSCs to aNSPCs and NB/IMNs (Fig. 2d and Extended Data Fig. 3j) that correlated with gene expression programs associated with maintenance of quiescence and stemness (for example, *Rfx4* and *Sox9*), NSC activation (for example, *Ascl1* and *Ybx1*) and subsequent differentiation into neuronal progeny (for example, *Neurod1* and *Sema5a*) (Extended Data Fig. 3k). Lineage progression was confirmed by graph edges representing shared nearest neighboring (SNN) cells that demonstrated the uni-directional lineage transition from qNSCs to aNSPCs and NB/IMNs (Fig. 2e).

qNSCs share several molecular markers with parenchymal astrocytes and have been assumed to undergo astrocytic transformation in the aging DG^39,40^. Thus, we analyzed if aging affects the molecular identity of qNSCs^41,42^. qNSCs and astrocytes occupied distinct UMAP spaces and displayed a robust number of DEGs (Extended Data Fig. 3l,m), indicative of distinct molecular profiles between these two populations. We classified gene expression patterns between qNSCs and astrocytes into three classes: genes expressed by astrocytes and qNSCs (that is, *Gfap* and *Sox2*); astrocyte-specific genes (that is, *S100b* and *Igfbp2*); and qNSC-specific genes (that is, *Ascl1* and *Stmn1*) (Fig. 2f and Extended Data Fig. 3n). To examine whether qNSCs acquire astrocytic identity with advanced aging, we analyzed the expression of the Ca^2+^-binding protein S100b, chosen from the astrocyte-specific gene set, to perform immunofluorescent staining using two independent mouse lines to genetically target NSCs, Nestin-GFP and Gli1CreERT2::R26-LSL-tdTOM^43–45^. NSCs were identified as GFP-expressing or tdTOM-expressing cells located in the subgranular zone (SGZ) with a single radial process oriented toward the molecular layer (ML). Across all three ages, NSCs were positive for both GFAP and SOX2 with only very few cells expressing S100b (Fig. 2g and Extended Data Fig. 3o). Thus, our data show that qNSCs retain their neurogenic potential and distinct molecular profiles compared to parenchymal astrocytes (despite stable S100b expression levels, astrocytic transformation of NSCs with old age cannot be ruled out).

### Neurogenic aging is a lifelong and multi-step process

**Fig. 3:**
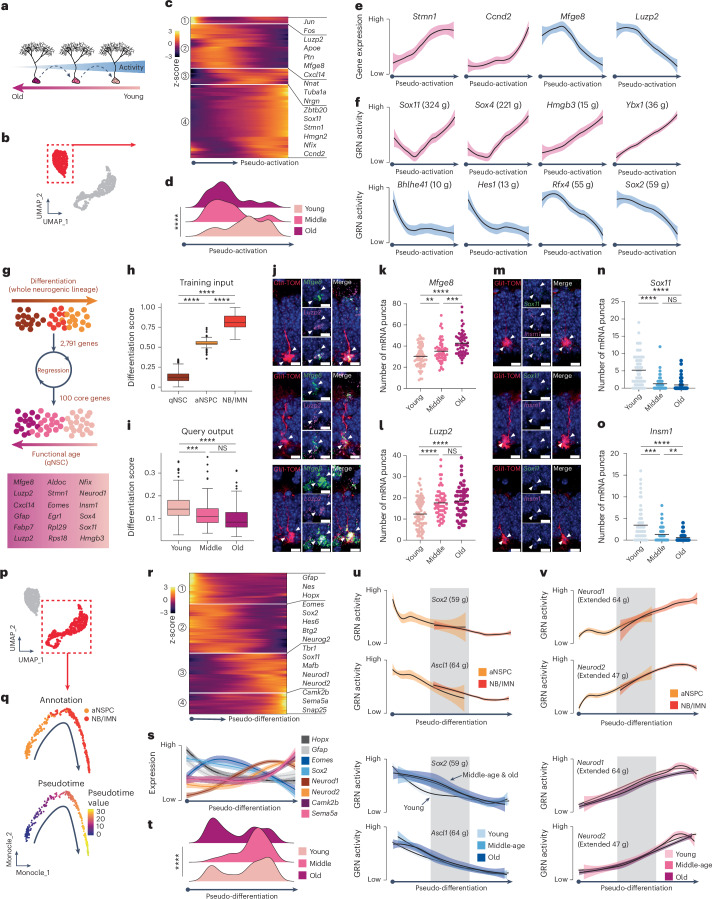
Neurogenic lineages undergo functional aging with biological aging. **a**, Working hypothesis of biological aging of qNSCs associated with functional aging. **b**, Selection of qNSCs from the whole neurogenic lineages. **c**, Heatmap shows gene expression dynamics of qNSCs across pseudotime. Numbers in circle indicate gene modules. Color gradient indicates the z-score of gene expression. **d**, Pseudotemporal ordering of qNSCs showing differential distribution along the pseudotemporal axis (one-way ANOVA, ****P < 0.0001). **e**, Pseudotemporal ordering of selected gene expression (Stmn1, Ccnd2, Mfge8 and Luzp2) related to neurogenic activation. Shading indicates 95% confidence interval. **f**, Pseudotemporal ordering of selected GRN activity from the Shin et al.^47^ TF list related to neurogenic activation (upper, upregulation: Sox11, Sox4, Hmgb3 and Ybx1; lower, downregulation: Bhlhe41, Hes1, Rfx4 and Sox2). Shading indicates 95% confidence interval. **g**–**i**, A random forest regression model was trained to predict the differentiated state of qNSCs from different ages. **g**, A core of 100 genes was identified in terms of neurogenic lineage progression state. **h**, Differentiation score of the whole neurogenic lineages as the training input (qNSC: 0.1308 ± 0.0042; aNSPC: 0.5490 ± 0.0049; NB/IMN: 0.8199 ± 0.0057; two-tailed unpaired *t*-test with Welch’s correction, ****P < 0.0001 between qNSC and aNSPC, ****P < 0.0001 between aNSPC and NB/IMN, ****P < 0.0001 between qNSC and NB/IMN). **i**, Differentiation score of qNSC as the query output (young: 0.1540 ± 0.0051; middle-age: 0.1235 ± 0.0072; old: 0.1044 ± 0.0079; two-tailed unpaired *t*-test with Welch’s correction, ***P = 0.0007 between young and middle-age, NS *P* = 0.0757 between middle-age and old, ****P < 0.0001 between young and old). Box plots depict the median and interquartile range, with whiskers indicating minimum and maximum values. **j**–**o**, In situ hybridization of candidate genes verifies the NAS. **j**, Representative images of RNAscope probes Mfge8 and Luzp2 together with labeling of radial NSCs by Gli1-CreERT2::tdTOM mouse line. **k**, Quantification of the number of Mfge8 puncta in radial NSCs (young: 30 ± 1 puncta; middle-age: 35 ± 1 puncta; old: 43 ± 1 puncta; two-tailed unpaired *t*-test with Welch’s correction, **P = 0.0089 between young and middle-age, ***P = 0.0003 between middle-age and old, ****P < 0.0001 between young and old). **l**, Quantification of the number of Luzp2 puncta in radial NSCs (young: 12 ± 1 puncta; middle-age: 18 ± 1 puncta; old: 18 ± 1 puncta; two-tailed unpaired *t*-test with Welch’s correction, ****P < 0.0001 between young and middle-age, NS *P* = 0.5875 between middle-age and old, ****P < 0.0001 between young and old). **m**, Representative images of RNAscope probes Sox11 and Insm1 together with labeling of radial NSCs by Gli1-CreERT2::tdTOM mouse line. **n**, Quantification of the number of Sox11 puncta in radial NSCs (young: 5 ± 1 puncta; middle-age: 1 ± 0 puncta; old: 1 ± 0 puncta; two-tailed unpaired *t*-test with Welch’s correction, ****P < 0.0001 between young and middle-age, NS *P* = 0.3867 between middle-age and old, ****P < 0.0001 between young and old). **o**, Quantification of the number of Insm1 puncta in radial NSCs (young: 3 ± 0 puncta; middle-age: 1 ± 0 puncta; old: 1 ± 0 puncta; two-tailed unpaired *t*-test with Welch’s correction, ***P = 0.0002 between young and middle-age, **P = 0.0088 between middle-age and old, ****P < 0.0001 between young and old). **p**, Selection of aNSPCs and NB/IMNs (fate-committed populations). **q**, Upper, visualization of monocle trajectories of the neuronal fate-committed populations; lower, projection of pseudotime value in the UMAP space. **r**, Heatmap shows gene expression dynamics of fate-committed populations across pseudotime. Numbers in circle indicate gene modules. Color gradient indicates the z-score of gene expression. **s**, Gene expression dynamics along pseudotemporal trajectory of selective genes representing putative active radial NSC (Hopx and Gfap), IPC (Eomes and Sox2), NB (Neurod1 and Neurod2) and IMN (Camk2b and Sema5a) stages. Shading indicates 95% confidence interval. **t**, Pseudotemporal ordering of fate-committed populations showing differential distribution along the pseudotemporal axis (one-way ANOVA, ****P < 0.0001). **u**,**v**, Projection of GRN activity in the pseudotemporal trajectory of selective TFs. Gray bar indicates putative transition zone between aNSPC and NB/IMN. **u**, GRN activity of Sox2 and Ascl1 along the pseudotemporal trajectory. Upper, visualization by the transition from aNSPC to NB/IMN; lower, visualization by individual ages. **v**, GRN activity of Neurod1 and Neurod2 along the pseudotemporal trajectory. Upper, visualization by the transition from aNSPC to NB/IMN; lower, visualization by individual ages. Shading indicates 95% confidence interval. Max, maximum; NS, not significant. All data are presented as mean ± s.e.m. Scale bars, 5 μm. Source data

**Extended Data Fig. 4:**
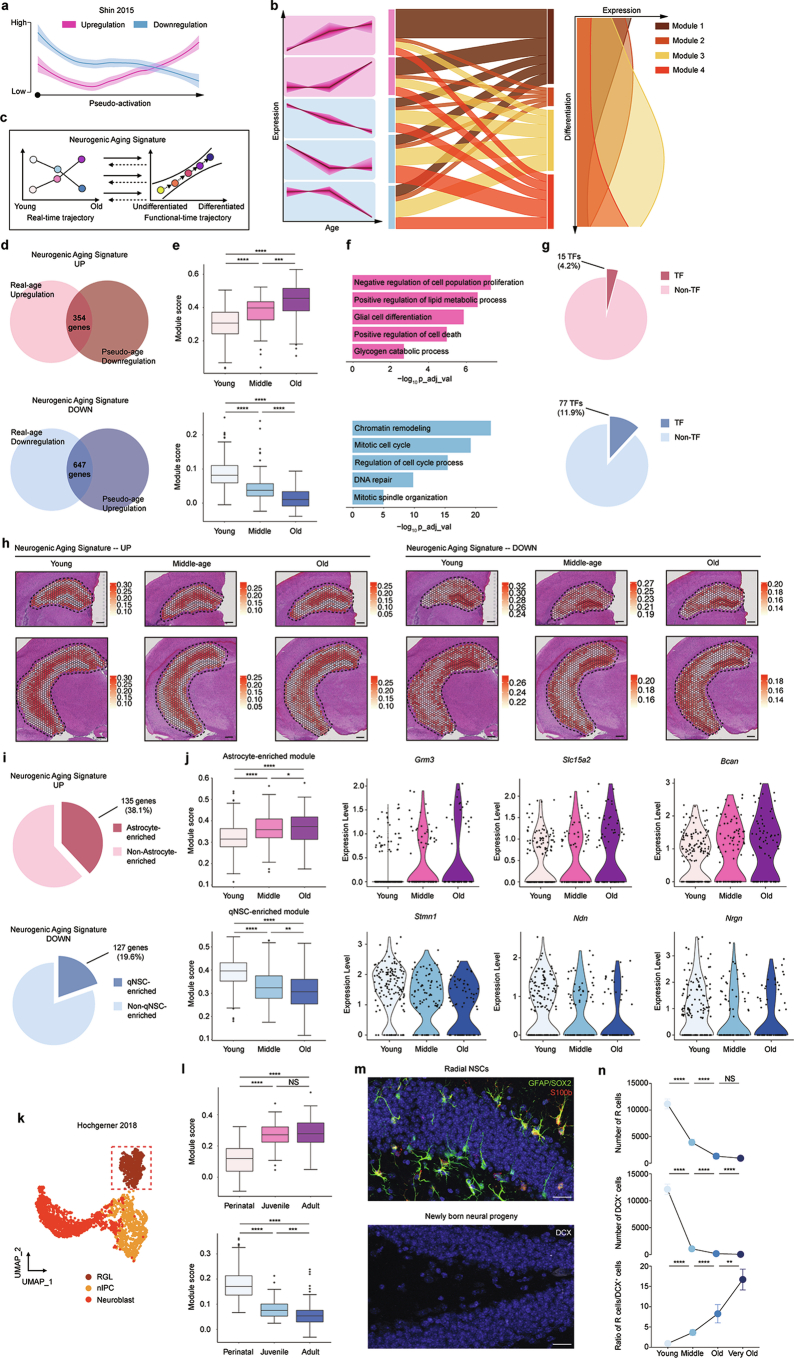
Continuous decrease in neurogenic activity is a hallmark of neurogenic aging. **a**, Projection of module score of Waterfall-defining TFs in pseudotemporal trajectory of qNSCs. Shading indicates 95% confidence interval. **b**, Alluvial plot between (left) real-time and (right) pseudotime gene expression trajectories of qNSC during aging shows age-dependent upregulation gene modules (in red) largely overlap with qNSC-enriched pseudotime gene modules while age-dependent downregulation gene modules (in blue) largely overlap with fate-committed population-enriched pseudotime gene modules. The line plots of the real-time gene expression trajectories depict the standardized gene expression signals, with each pink line representing the signal of the same gene in different cell populations and the black lines indicating the mean values for each gene cluster. **c**, Schematic overview of the Neurogenic Aging Signature calculation. **d**, Venn diagram indicating the overlap between the real-time and pseudotime trajectories. Upper: Between Real-age (real-time) upregulation and functional-age (pseudotime) downregulation; Lower: Between Real-age (real-time) downregulation and functional-age (pseudotime) upregulation. **e**, Module scores of Neurogenic Aging Signature of qNSCs. Upper: Neurogenic Aging Signature-UP score of qNSCs (two-tailed unpaired t-test with Welch’s correction; t = 6.217, ****P < 0.0001 between young and middle-age, t = 3.739, ***P = 0.0003 between middle-age and old, t = 9.396, ****P < 0.0001 between young and old; young, n = 141 cells, middle-age, n = 84 cells, old, n = 69 cells). Lower: Neurogenic Aging Signature-DOWN score of qNSCs (two-tailed unpaired t-test with Welch’s correction; t = 6.843, ****P < 0.0001 between young and middle-age, t = 4.930, ****P < 0.0001 between middle-age and old, t = 13.55, ****P < 0.0001 between young and old; young, n = 141 cells, middle-age, n = 84 cells, old, n = 69 cells). Box plots depict the median and interquartile range, with whiskers indicating minimum and maximum values. **f**, GO terms enriched in (upper) Neurogenic Aging Signature-UP and (lower) Neurogenic Aging Signature-DOWN. All GO terms shown by an adjusted *P* < 0.05 with Benjamini–Hochberg correction. **g**, Pie charts of the percentage of TFs in Neurogenic Aging Signature (upper) -UP and (lower) -DOWN. **h**, Spatial feature plots of (left) Neurogenic Aging Signature-UP and (right) Neurogenic Aging Signature-DOWN showing regional-specific expression patterns in hippocampus. Color gradient indicates the expression level of individual module score. **i**, Venn diagram indicating the overlap between (upper) the Astrocyte-enriched genes and Neurogenic Aging Signature-UP and (lower) the qNSC-enriched genes and Neurogenic Aging Signature-DOWN. **j**, Expression of Astrocyte- and qNSC-enriched genes in qNSCs among different ages. Upper: Module scores of the Astrocyte-enriched genes in qNSC (two-tailed unpaired t-test with Welch’s correction; t = 5.241, ****P < 0.0001 between young and middle-age, t = 2.462, *P = 0.0151 between middle-age and old, t = 7.172, ****P < 0.0001 between young and old; young, n = 141 cells, middle-age, n = 84 cells, old, n = 69 cells) and expression of selected Astrocyte-enriched genes, *Grm3*, *Slc15a2* and *Bcan*. Lower: Module scores of the qNSC-enriched genes in qNSC (two-tailed unpaired t-test with Welch’s correction; t = 5.035, ****P < 0.0001 between young and middle-age, t = 3.037, **P = 0.0028 between middle-age and old, t = 8.787, ****P < 0.0001 between young and old; young, n = 141 cells, middle-age, n = 84 cells, old, n = 69 cells). Box plots depict the median and interquartile range, with whiskers indicating minimum and maximum values. **k**, UMAP plot of neurogenic lineages in Hochgerner 2018 dataset. **l**, Module scores of Neurogenic Aging Signature of RGL. Upper: Neurogenic Aging Signature-UP score of RGL (two-tailed unpaired t-test with Welch’s correction; t = 12.79, ****P < 0.0001 between perinatal and juvenile, t = 1.098, NS, *P* = 0.2745 between juvenile and adult, t = 9.396, ****P < 0.0001 between perinatal and adult; perinatal, n = 461 cells, juvenile, n = 57 cells, adult, n = 127 cells). Lower: Neurogenic Aging Signature-DOWN score of RGL (two-tailed unpaired t-test with Welch’s correction; t = 16.10, ****P < 0.0001 between perinatal and juvenile, t = 3.501, ***P = 0.0007 between juvenile and adult, t = 25.22, ****P < 0.0001 between perinatal and adult; perinatal, n = 461 cells, juvenile, n = 57 cells, adult, n = 127 cells). Box plots depict the median and interquartile range, with whiskers indicating minimum and maximum values. **m**, Immunofluorescent staining of radial NSCs (GFAP^+^SOX2^+^S100b^−^ cells with radial morphology) and newly born neuronal progeny (DCX^+^ cells). **n**, Quantification of (upper) the number of radial NSC (young: 11151 ± 375 cells; middle-age: 3923 ± 200 cells; old: 1324 ± 154 cells; very old: 934 ± 100 cells; two-tailed unpaired t-test with Welch’s correction, ****P < 0.0001 between young and middle-age, ****P < 0.0001 between middle-age and old, NS, *P* = 0.0558 between old and very old), (middle) the number of neuroblast (young: 12131 ± 392 cells; middle-age: 1079 ± 32 cells; old: 173 ± 25 cells; two-tailed unpaired t-test with Welch’s correction, ****P < 0.0001 between young and middle-age, ****P < 0.0001 between middle-age and old, ****P < 0.0001 between old and very old) and (lower) the ratio of radial NSCs to neuroblasts from young (3 MO) to very old (24-25 MO) (young: 0.9 ± 0.0; middle-age: 3.7 ± 0.2; old: 8.3 ± 0.7; very old: 16.7 ± 1.3; two-tailed unpaired t-test with Welch’s correction, ****P < 0.0001 between young and middle-age, ****P < 0.0001 between middle-age and old, ****P < 0.0001 between old and very old). aNSPC, active neural stem and progenitor cells, GO, gene ontology, GRN, gene regulatory network, IPC, intermediate progenitor cell, MO, month-old, NB/IMN, neuroblast and immature neuron, NS, not significant, NSC, neural stem cell, SGZ, subgranular zone, TF, transcription factor. All data were presented as mean ± SEM. Scale bars, 20 μm. Source data

Adult neurogenesis is a multi-step process, consisting of the activation of qNSCs and differentiation of their fate-committed progeny^11,46^. We previously used intravital imaging to define age-dependent cellular alterations of hippocampal neurogenesis and found, in accordance with previous snapshot-based analyses^22,23^, deeper quiescence, a more frequent return to long-term quiescence and extended cell division length^47^. To identify molecular mechanisms of how aging affects distinct cell types of the neurogenic lineage (hereafter referred to as neurogenic aging), we performed pseudotime trajectory analysis of qNSCs and their fate-committed progeny. First, we selected the qNSC cluster from all analyzed timepoints and recalculated their pseudotime values to construct the qNSC-only trajectory (Fig. 3a–c). However, it is important to note that the unbiased isolation approach used here does not allow to distinguish long-term self-renewing versus more exhausting qNSC pools^23,45^. The qNSC trajectory displayed age-dependent differential distribution (Fig. 3d) and mirrored gene expression dynamics of the whole neurogenic trajectory (including qNSC, aNSPC and NB/IMN): astrocytic/progenitor genes (for example, *Mfge8* and *Luzp2*) were downregulated and cell cycle and neuronal genes (for example, *Ccnd2* and *Stmn2*) were upregulated along the qNSC trajectory (Fig. 3e). A molecular cascade of TFs underlying neurogenic activation was previously identified, showing that a list of TFs for the maintenance of quiescence is highly expressed in qNSCs, whereas another list of TFs for activation and neurogenesis is upregulated in aNSPCs^48^. Indeed, we first curated and mapped these two lists of TFs onto the qNSC trajectory to validate that the expression of TFs upregulated and downregulated upon qNSC activation increased and decreased along the qNSC trajectory, respectively (Extended Data Fig. 4a). Then, we further found that the qNSC trajectory across the lifespan validated the expression dynamics of this molecular cascade where qNSCs from advancing ages remained in deeper quiescence compared to younger qNSCs (Fig. 3d,f). Thus, we refer to the qNSC trajectory as the pseudo-activation trajectory. This result was further supported by a random forest regression model in which we took the whole neurogenic lineage as the training dataset to query the qNSC population and found a clear difference between young and aged qNSCs in terms of their differentiation state (Fig. 3g–i). Thus, our findings identify molecular changes in aging qNSCs that may underlie previously characterized functional alterations of qNSC behavior^47^.

One major limitation of pseudotime-based trajectory analyses is that they do not necessarily report the same changes that would be observed with real-time progression^49^. To overcome this, we constructed the real-time gene expression trajectory (Methods) to examine the intersection between real-time and pseudotime trajectories (Extended Data Fig. 4b). In brief, we first used soft clustering to identify the temporal trajectory of gene expression signatures ordered along biological ages (for example, from young to middle-age and old; Supplementary Table 3). This clustering approach allows any data point to have more than one cluster label, offering the flexibility for the nuanced representation of imprecise data^50^. We then intersected gene modules ordered in real-time series with gene modules identified using the Monocle algorithm. Indeed, the real-time upregulation trajectory largely overlapped with the qNSC-enriched pseudotime module (module 1), whereas the real-time downregulation trajectory largely overlapped with fate-committed population-enriched pseudotime modules (modules 3 and 4) (Extended Data Fig. 4b). These data further suggest that qNSCs undergo molecular changes with advancing age in terms of their activation and differentiation states, identifying pathways that may be targeted in future experiments to ameliorate age-dependent changes of qNSCs.

Gene sets that detect the quiescent state of NSCs at different ages are currently missing. Thus, we generated a gene signature, hereafter referred to as Neurogenic Aging Signature (NAS), by selecting genes overlapping between the real-time and pseudotime modules to reflect both real-time and pseudotime changes in qNSCs (Extended Data Fig. 4c,d and Methods). We found that qNSCs from different ages could indeed be ordered by both upregulation and downregulation signatures, suggesting that qNSCs undergo constant age-dependent molecular changes (Extended Data Fig. 4e,f). Interestingly, the proportion of TFs was higher in the downregulation signature compared to the upregulation signature, indicating that reduced ability of activation might represent a key property of neurogenic aging (Extended Data Fig. 4g)^14,51^. Moreover, using ST, we noticed that the NAS upregulation signature was constitutively expressed in the ML, whereas the NAS downregulation signature was constitutively expressed in the neuronal layer of hippocampus, in line with their functional annotation that the upregulation and downregulation signatures are mostly related to glia-related and neuronal-related functions (Extended Data Fig. 4f,h). These results were in line with their functional annotation that the upregulation and downregulation signatures largely overlapped with astrocyte-enriched and qNSC-enriched genes, respectively. We further confirmed it by dissecting astrocyte-enriched and qNSC-enriched genes (Extended Data Fig. 3m) that largely overlapped with the upregulation and downregulation signatures, respectively, and showed age-dependent expression changes (Extended Data Fig. 4i,j). Our results highlight that qNSCs enhance their molecular features of the glial identity, whereas their gene expression features related to neuronal differentiation decrease with age.

We validated the NAS using RNA in situ hybridization in Gli1CreERT2::R26-LSL-tdTOM mice targeting qNSCs. We analyzed the expression of *Mfge8* and *Luzp2* from the NAS upregulation signature and *Sox11* and *Insm1* from the NAS downregulation signature and verified their age-dependent regulation of expression (Fig. 3j–o). Furthermore, we tested the NAS using a published dataset that comprises three early developmental stages—perinatal (embryonic (E) day 16.5 to postnatal (P) day 5), juvenile (P18–P23) and young adult (4 MO)^34^—and found that, even at earlier ages, qNSCs showed similar age-dependent relative changes in the NAS value, further demonstrating that hippocampal NSCs undergo continuous changes from early development to adulthood to old age^34^ (Extended Data Fig. 4k,l). Indeed, qNSCs could still be identified in the 2-year-old mouse DG but with very limited neurogenic output (Extended Data Fig. 4m,n). Our findings provide insights into the age-dependent decrease of hippocampal neurogenesis that appear to be mainly driven by a constant decrease in gene expression profiles required for qNSC activation.

**Extended Data Fig. 5:**
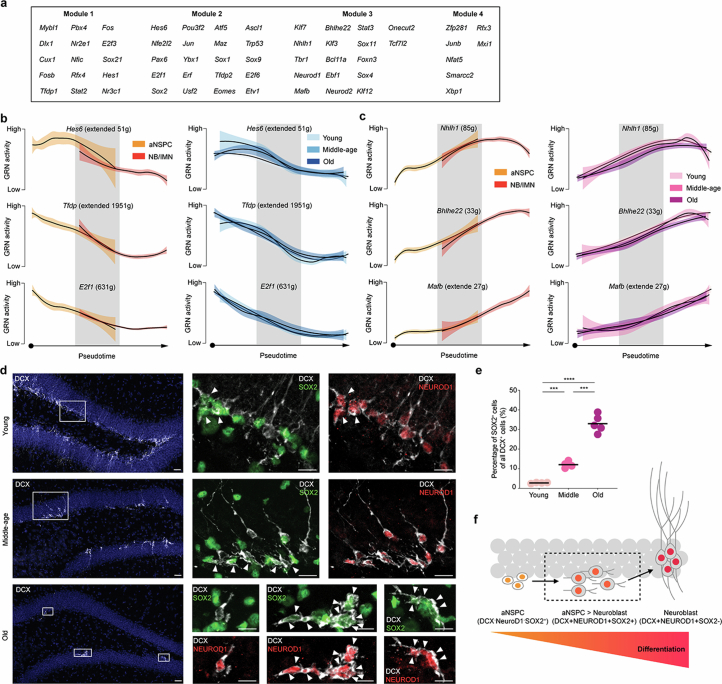
Impaired lineage progression of fate-committed neural progeny with aging. **a**, List of TFs in each pseudotime modules of which the GRN have been successfully reconstructed. **b-c**, Projection of GRN activity in the pseudotemporal trajectory of addtional transcription factors. Grey bar indicates putative transition zone between aNSPC and NB/IMN. (**b**) GRN activity of Hes6, Tfdp and E2f1 along the pseudotemporal trajectory. Left: Visualization by the transition from aNSPC to NB/IMN. Right: Visualization by individual ages. (**c**) GRN activity of Nhlh, Bhlhe22 and Mafb along the pseudotemporal trajectory. Upper: Visualization by the transition from aNSPC to NB/IMN. Lower: Visualization by individual ages. Shading indicates 95% confidence interval. Gray zone indicates the transition from aNSPC to NB/IMN. **d**, Immunofluorescent staining of SOX2 and neuroblasts (NEUROD1 and DCX) showing this transitional stage of neural progeny differentiation is delayed in advancing ages. **e**, Quantification of the percentage of SOX2^+^ neuroblasts among all neuroblasts (NEUROD1^+^DCX^+^) at different ages (young: 2.8 ± 0.1%; middle-age: 12.1 ± 0.8%; old: 33.0 ± 2.0%; two-tailed unpaired t-test with Welch’s correction, **P = 0.0012 between young and middle-age, ***P = 0.0001 between middle-age and old, ****P < 0.0001 between young and old). **f**, Schematic illustration of delayed lineage progression from aNSPC to NB/IMN. aNSPC, active neural stem and progenitor cells, GRN, gene regulatory network and NB/IMN, neuroblast and immature neuron. All data were presented as mean ± SEM. Scale bars, 20 μm. Source data

After cell cycle entry, neurogenic cells undergo fate commitment and eventually give rise to granule cells (GCs). We and others previously revealed that aging strongly affects the differentiation and maturation of neuronal progeny, but little is known about the effects of aging on molecular mechanisms regulating neural differentiation^47,52^. We analyzed the molecular programs regulating neural differentiation of fate-committed neural progeny (Fig. 3p) and re-clustered aNSPCs and NB/IMNs after regressing out cell cycle genes to remove the influence of cell cycle state on cell differentiation. Cells were ordered into a single trajectory (Fig. 3q,r), in which gene modules related to radial NSC identity (module 1—for example, *Gfap* and *Hopx*), intermediate progenitor cell (IPC) identity (module 2—for example, *Eomes* and *Sox2*), neuronal specification (module 3—for example, *Neurod1* and *Neurod2*) and maturation (module 4—for example, *Camk2b* and *Sema5a*) were sequentially expressed, reflecting putative stages of active NSCs and IPCs (collectively termed aNSPCs) as well as neuroblasts and immature neurons (collectively termed NB/IMNs) (Fig. 3s). Notably, different ages showed differential distribution along the pseudotime axis, correlating with the transition from a progenitor state (module 2) to a neuronal state (module 3) (Fig. 3t). Neuronal differentiation requires the orchestration of TF regulatory networks to transition from progenitor/glial to neuronal cell fate^53^. We speculated that the TF regulatory networks for the transition from progenitor state (module 2) to neuronal identity (module 3) could be altered with advancing age. Thus, we constructed GRNs of TFs and investigated their dynamics along a pseudotime trajectory. In total, we constructed GRNs of 37 TFs among all 112 TFs in modules 2 and 3 (Extended Data Fig. 5a and Supplementary Table 4). Interestingly, select TFs in module 2 (for example, *Sox2* and *Hes6*) displayed a lag of downregulation in aged lineages, whereas many other TFs of module 2 shared similar temporal dynamics (for example, *Neurod1* and *Neurod2*) (Fig. 3u,v and Extended Data Fig. 5b,c). This finding was confirmed using protein expression analyses of newborn neuronal progeny (DCX^+^NEUROD1^+^) co-stained with SOX2 where we found that the fraction of SOX2^+^ newborn neuronal progeny strongly increased with age (Extended Data Fig. 5d,e). Thus, our results identify altered progression from the progenitor state to a committed neuronal, GC identity in the aged hippocampus (Extended Data Fig. 5f).

Together, our data reveal that aging impairs the activation of qNSCs, as suggested previously in the DG of younger mice^22,23^, but also affects lineage progression of fate-committed neural progeny. Age-dependent molecular alterations affect the entire neurogenic lineage, from qNSCs to immature neurons, that are detectable at middle-age but become more pronounced in the aged DG.

### Age-dependent changes of the neurogenic niche in the DG

The DG stem cell niche provides a permissive environment allowing to respond to stimuli causing the activation of qNSCs and subsequent maturation of their neuronal progeny^54,55^; however, little is known about molecular alterations of the hippocampal niche during aging^11,55^. Thus, we characterized the expression profiles of main hippocampal niche populations—astrocytes, vasculature and microglia—that have been reported to play critical roles not only in the regulation of neurogenesis but also in various neurodegenerative diseases^11,56–59^.

**Fig. 4:**
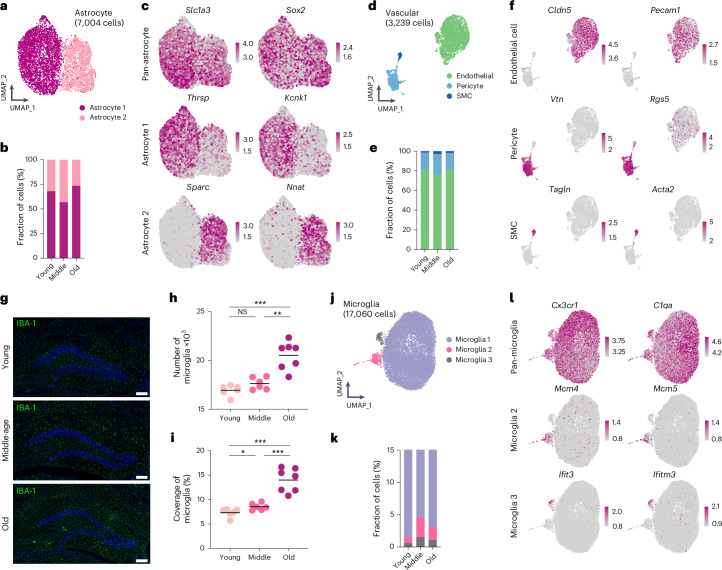
Age-dependent changes of main populations in the mouse DG. **a**, UMAP visualization of the heterogeneity of astrocytes. **b**, Composition of each astrocyte subpopulation in the mouse DG. **c**, Selected expression of subtype-specific genes of astrocytes. Color gradient indicates the log-normalized gene expression level. **d**, UMAP visualization of the entire vascular compartment. **e**, Composition of each cell type within the vascular compartment in the mouse DG. **f**, Selective expression of cell-type-specific genes of the vascular compartment. Color gradient indicates the log-normalized gene expression level. **g**, Representative images of IBA-1^+^ microglia in the DG of young (upper), middle-aged (middle) and old (lower) mice showing increased number and signal coverage with aging. **h**,**i**, Quantification of the number of IBA-1^+^ microglia (young: 16,940 ± 221.0 cells; middle-age: 17,636 ± 250.7 cells; old: 20516 ± 536.5 cells; two-tailed unpaired *t*-test with Welch’s correction, NS *P* = 0.0646 between young and middle-age, **P = 0.0011 between middle-age and old, ***P = 0.0003 between young and old) (**h**) and the coverage of IBA-1 signal in the DG (young: 7.317 ± 0.3516%; middle-age: 8.550 ± 0.2837%; old: 14.01 ± 0.9075%; two-tailed unpaired *t*-test with Welch’s correction, *P = 0.0220 between young and middle-age, ***P = 0.0006 between middle-age and old, ***P = 0.0001 between young and old) (**i**). **j**, UMAP visualization of the heterogeneity of microglia. **k**, Relative proportions of each subtype of microglia in the mouse DG. **l**, Selective expression of subtype-specific genes of different microglia subpopulations in the UMAP space. Color gradient indicates the log-normalized gene expression level. NS, not significant; SMC, smooth muscle cell. All data are presented as mean ± s.e.m. Scale bars, 100 μm. Source data

**Extended Data Fig. 6:**
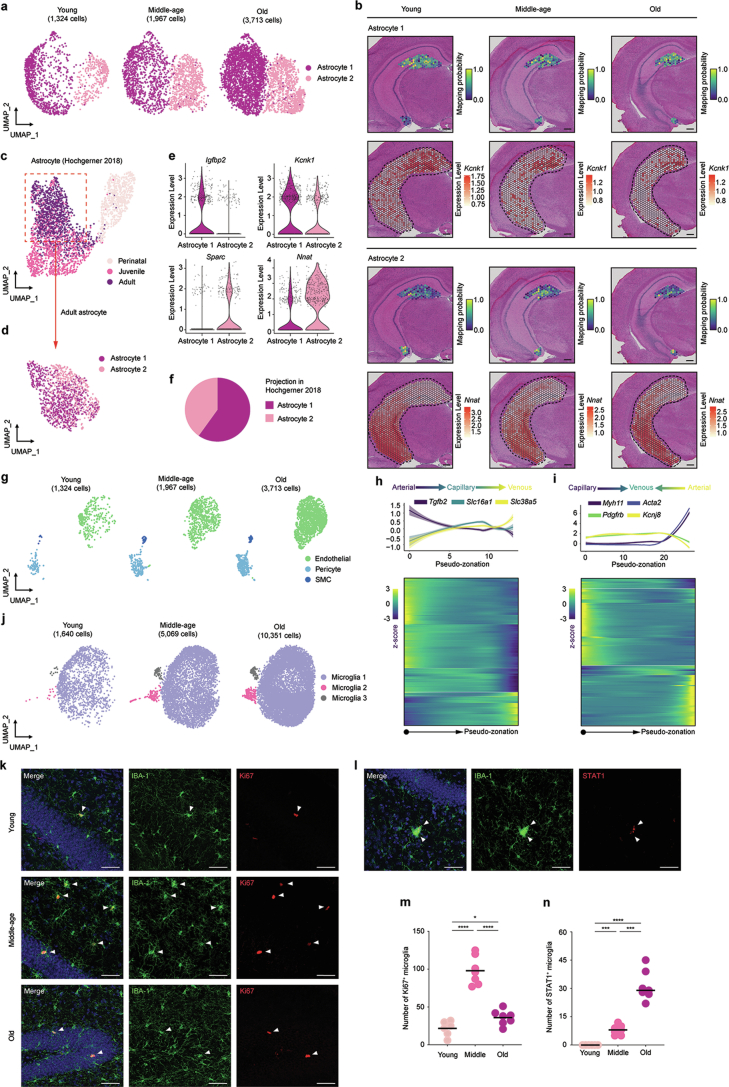
Characterization of main populations in the neurogenic niche with advancing age. **a**, UMAP visualization of astrocyte subtypes in (left) Young, (middle) Middle-age and (right) Old mice. **b**, Spatial distribution of astrocyte subtypes among different ages using tangram cell type mapping. Estimated mapping probability of each astrocyte subtype (color gradient) to DG shown over the H&E images. **c**, UMAP visualization of astrocyte from Hochgerner 2018 dataset. **d**, Projection of predicted identities of Astrocyte 1 and Astrocyte 2 in adult astrocytes of Hochgerner 2018 dataset. **e**, Violin plots showing similar expression of Astrocyte 1- (Igfbp2 and Kcnk1) and 2-specific (Sparc and Nnat) genes in adult astrocytes of Hochgerner et al.^34^ dataset. **f**, Pie chart showing the composition of Astrocyte 1 and Astrocyte 2 of adult astrocytes in Hochgerner 2018 dataset (Astrocyte 1: 59.8% and Astrocyte 2: 40.2%). **g**, UMAP visualization of vascular cell types in (left) Young, (middle) Middle-age and (right) Old mice. **h-i**, Molecular zonation of vascular compartment. Lower: Smoothened heat map of molecular patterns of the zonation of (**h**) endothelial and (**i**) mural cells. Gene expression was ordered based on the peak expression level along the pseudotemporal axis. Color gradient indicates the z-score of gene expression. Upper: Projection of gene expression dynamics of selective genes in pseudotemporal trajectory representing (**h**) endothelial zonation (Tgfb2, Slc16a1 and Slc38a5) and (**i**) mural zonation (Myh11, Acta2, Pdgfrb and Kcnj8). Color gradient indicates the log-normalized gene expression level. Shading indicates 95% confidence interval. **j**, UMAP visualization of microglia subtypes in (left) Young, (middle) Middle-age and (right) Old mice. **k**, Representative images of proliferating microglia (Microglia 2, indicated by arrowhead) co-stained with Ki67 in DG of (upper) young, (middle) middle-age and (lower) old mice. **l**, Representative images of inflammation-responding microglia (Microglia 3, indicated by arrowhead) in SGZ of old mice. **m-n**, Quantification of microglia subpopulations in SGZ at different ages. (**m**) Quantification of the number of Ki67^+^ microglia in SGZ (young: 22 ± 4 cells; middle-age: 98 ± 7 cells; old: 36 ± 4 cells; two-tailed unpaired t-test with Welch’s correction, ****P < 0.0001 between young and middle-age, ****P < 0.0001 between middle-age and old, *P = 0.0233 between young and old). (**n**) Quantification of the number of STAT1^+^ microglia in SGZ (young: 0 ± 0 cells; middle-age: 8 ± 1 cells; old: 31 ± 3 cells; two-tailed unpaired t-test with Welch’s correction, ***P = 0.0002 between young and middle-age, ***P = 0.0001 between middle-age and old, ****P < 0.0001 between young and old). UMAP, uniform manifold approximation and projection. All data were presented as mean ± SEM. Scale bars, (**b**) 200 μm and (**k** and **l**) 20 μm. H&E, Haematoxylin and eosin. Source data

We observed two molecularly distinct subpopulations of astrocytes, hereafter referred to as Astro 1 and Astro 2 (Fig. 4a–c and Extended Data Fig. 4). All astrocytes highly expressed pan-astrocytic markers (for example, *Gfap*, *S100b* and *Hopx*) (Figs. 1d and 4c). Astrocyte subpopulations expressed distinct sets of genes (for example, Astro 1: *Kcnk1* and *Thrsp*; Astro 2: *Sparc* and *Nnat*) (Fig. 4c). The relative proportion of the two astrocyte subpopulations, with most representing Astro 1, remained stable across the adult lifespan (Fig. 4b and Extended Data Fig. 6a). Notably, deconvoluted ST data revealed regional heterogeneity across astrocyte subpopulations: Astro 1 mainly distributed in the dorso-medial DG, whereas Astro 2 was enriched in the ventro-lateral DG (Extended Data Fig. 6b). Interestingly, this regional pattern mirrored two subtype-specific genes, *Kcnk1* of Astro 1 and *Nnat of* Astro 2, indicating potential differential functions across different hippocampal subregions. To validate our findings, we re-analyzed a previously published dataset profiling the mouse DG and were able to project Astro 1 and Astro 2 identities onto adult astrocytes that have been described using scRNA-seq^34^ (Extended Data Fig. 6c–f). Thus, astrocyte populations remain stable in the aging DG.

The vascular compartment comprises three main cell types—endothelial cells, pericytes and smooth muscle cells (together known as mural cells)—that are essential for the formation of the blood–brain barrier (BBB) in the central nervous system^60^. To examine their molecular profiles and abundances, we re-clustered the three vascular populations (Fig. 4d–f). The relative fraction of each cell type remained similar at each age, indicating a stable cellular composition of the vascular network throughout lifetime (Fig. 4e). Previous work showed molecular zonation of vascular cells in the central nervous system, with cellular phenotypes gradually changing along an anatomical axis^59,61^. In line with these studies, we observed that both endothelial and mural cells displayed clear molecular zonation based on gene expression profiles fitting an anatomical axis (hereafter called pseudo-zonation), although the cellular diversity within each cell type of the whole vascular compartment was limited (Extended Data Fig. 6f,g).

Microglia are brain resident immune cells that are essential for the maintenance of neurogenic cells^62^. Notably, the proportion of microglia increased with advancing age as judged by relative sequencing frequency (Fig. 1e), which we confirmed by measuring the number and IBA-1 coverage in the aged DG using immunohistochemistry (Fig. 4g–i). After re-clustering, we identified three subpopulations: Microglia 1, 2 and 3 (Fig. 4j). Unlike astrocytes or cells of the vasculature, microglial cells showed a selective enrichment of age-dependent subpopulations, with an increase in relative proportions of Microglia 2 and Microglia 3 (Fig. 4k). In addition to pan-microglia markers, such as *Cx3cr1* and *C1qa*, Microglia 2 preferentially expressed cell cycle genes (for example, *Mcm4* and *Mcm5*), and Microglia 3 showed an upregulation of genes related to various inflammatory responses (for example, *Ifit3* and *Ifitm3*) (Fig. 4l), representing putative proliferative and inflammatory subpopulations, respectively. We confirmed the age-dependent change in microglia subpopulations (that is, Microglia 2 and 3) by using immunofluorescent staining against the microglia markers IBA-1, Ki67 and STAT1 (Extended Data Fig. 6k–n), in line with previous work that analyzed alterations of microglia in the aging mouse brain^63^.

Although the cellular composition of astrocytes and cells of the vasculature remains relatively stable during aging, there is an increase in both the number and diversity of microglia in the aged DG. Given that microglia play a pivotal role in the immune surveillance during aging and degenerative diseases^64,65^, we further investigated the global cues of the entire DG niche and their relevance to neurogenic aging.

### Core Aging Signature in the aging DG

**Fig. 5:**
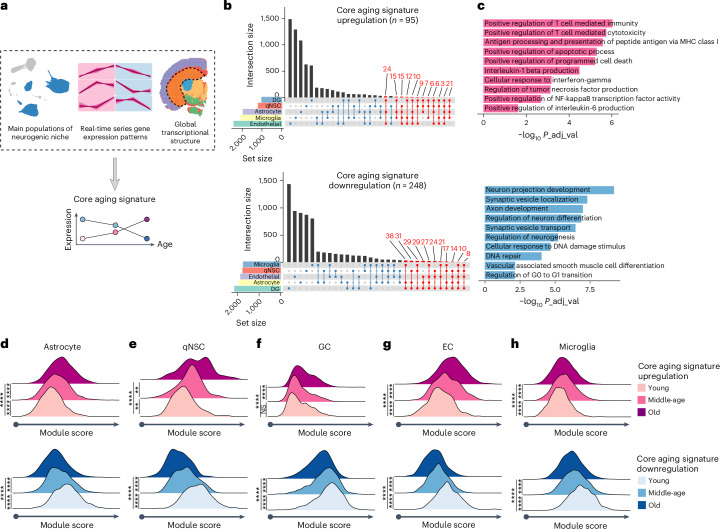
CAS with advancing age. **a**, Schematic overview of calculation of the module score of the CAS. **b**, Upset plots showing cell-type-specific and shared DEGs between main niche populations and the global transciptome of DG. Upper, age-dependent upregulation DEGs; lower, age-dependent downregulation DEGs. **c**, GO terms enriched in CAS-Up (upper) and CAS-Down (lower) score. All GO terms are shown by an adjusted *P* < 0.05 with Benjamini–Hochberg correction. **d**–**h**, CAS scores in astrocyte (two-tailed unpaired *t*-test with Welch’s correction; CAS-Up: *t* = 13.02, ****P < 0.0001 between young and middle-age; *t* = 13.58, ****P < 0.0001 between middle-age and old; *t* = 27.40, ****P < 0.0001 between young and old; CAS-Down: *t* = 15.99, ****P < 0.0001 between young and middle-age; *t* = 11.26, ****P < 0.0001 between middle-age and old; *t* = 27.12, ****P < 0.0001 between young and old) (**d**); qNSC (two-tailed unpaired *t*-test with Welch’s correction; CAS-Up: *t* = 3.250, **P = 0.0014 between young and middle-age; *t* = 2.913, **P = 0.0042 between middle-age and old; *t* = 5.698, ****P < 0.0001 between young and old; CAS-Down: *t* = 6.968, ****P < 0.0001 between young and middle-age; *t* = 4.121, ****P < 0.0001 between middle-age and old; *t* = 11.29, ****P < 0.0001 between young and old) (**e**); GC (two-tailed unpaired *t*-test with Welch’s correction; CAS-Up: *t* = 1.371, NS *P* = 0.1705 between young and middle-age; *t* = 9.256, ****P < 0.0001 between middle-age and old; *t* = 13.98, ****P < 0.0001 between young and old; CAS-Down: *t* = 8.109, ****P < 0.0001 between young and middle-age; *t* = 9.527, ****P < 0.0001 between middle-age and old; *t* = 24.75, ****P < 0.0001 between young and old) (**f**); EC (two-tailed unpaired *t*-test with Welch’s correction; CAS-Up: *t* = 8.506, ****P < 0.0001 between young and middle-age; *t* = 7.423, ****P < 0.0001 between middle-age and old; *t* = 15.50, ****P < 0.0001 between young and old; CAS-Down: *t* = 13.06, ****P < 0.0001 between young and middle-age; *t* = 9.099, ****P < 0.0001 between middle-age and old; *t* = 20.92, ****P < 0.0001 between young and old) (**g**); and microglia (two-tailed unpaired *t*-test with Welch’s correction; CAS-Up: *t* = 17.23, ****P < 0.0001 between young and middle-age; *t* = 4.486, ****P < 0.0001 between middle-age and old; *t* = 15.61, ****P < 0.0001 between young and old; CAS-Down: *t* = 18.94, ****P < 0.0001 between young and middle-age; *t* = 17.16, ****P < 0.0001 between middle-age and old; *t* = 31.36, ****P < 0.0001 between young and old) (**h**). Upper, ridge plot showing CAS-Up; lower, ridge plot showing CAS-Down score in each cell type. qNSC, quiescent neural stem cell; GC, granule cell; EC, endothelial cell; NS, not significant. Source data

**Extended Data Fig. 7:**
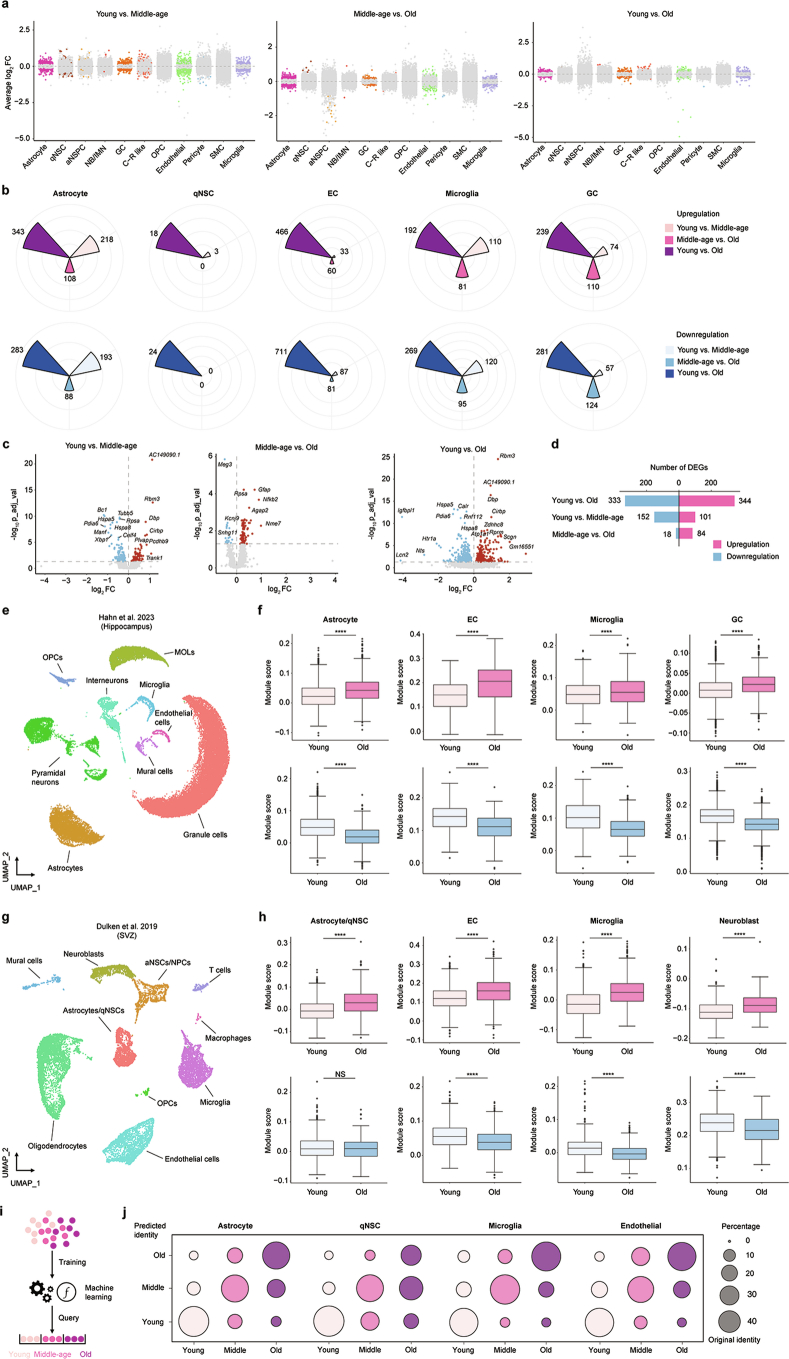
Pairwise comparison and regression model of age-dependent differential gene expression profile. **a**, Dot plot showing the age-dependent changes for each cell types in dentate gyrus between (left) young and middle-age, (middle) middle-age and old, and (right) young and old. For all dots shown in color by an adjusted *P* < 0.05 with Bonferroni correction, MAST differential test. **b**, Wind rose plots showing the number of DEGs of selected cell types in dentate gyrus (upper) upregulated and (lower) downregulated between young and middle-age, middle-age and old, and young and old. **c**, Volcano plots showing DEGs between each age of the entire DG derived from the ST data. **d**, Bar plot shows the number of DEGs corresponding to **c**. **e**, UMAP plot of all cell types in Hahn 2023 dataset. **f**, CAS scores in Astrocyte (CAS-Up: two-tailed unpaired t-test with Welch’s correction, t = 17.21, ****P < 0.0001 between young and old; CAS-Down: two-tailed unpaired t-test with Welch’s correction, t = 29.91, ****P < 0.0001 between young and old; young, n = 2,787 cells, old, n = 1,927 cells), EC (CAS-Up: two-tailed unpaired t-test with Welch’s correction, t = 7.604, ****P < 0.0001 between young and old; CAS-Down: two-tailed unpaired t-test with Welch’s correction, t = 6.703, ****P < 0.0001 between young and old; young, n = 229 cells, old, n = 178 cells), Microglia (CAS-Up: two-tailed unpaired t-test with Welch’s correction, t = 27.97, ****P < 0.0001 between young and old; CAS-Down: two-tailed unpaired t-test with Welch’s correction, t = 11.59, ****P < 0.0001 between young and old; young, n = 526 cells, old, n = 336 cells) and GC (CAS-Up: two-tailed unpaired t-test with Welch’s correction, t = 33.66, ****P < 0.0001 between young and old; CAS-Down: two-tailed unpaired t-test with Welch’s correction, t = 59.63, ****P < 0.0001 between young and old; young, n = 13,516 cells, old, n = 6,033 cells) in Hahn 2023 dataset. Box plots depict the median and interquartile range, with whiskers indicating minimum and maximum values. **g**, UMAP plot of all cell types in Dulken 2019 dataset. **h**, CAS scores in Astrocyte/qNSC (CAS-Up: two-tailed unpaired t-test with Welch’s correction, t = 12.35, ****P < 0.0001 between young and old; CAS-Down: two-tailed unpaired t-test with Welch’s correction, t = 1.830, NS, *P* = 0.0676 between young and old; young, n = 1,103 cells, old, n = 476 cells), EC (CAS-Up: two-tailed unpaired t-test with Welch’s correction, t = 15.00, ****P < 0.0001 between young and old; CAS-Down: two-tailed unpaired t-test with Welch’s correction, t = 13.18, ****P < 0.0001 between young and old; young, n = 1,389 cells, old, n = 1,355 cells), Microglia (CAS-Up: two-tailed unpaired t-test with Welch’s correction, t = 21.22, ****P < 0.0001 between young and old; CAS-Down: two-tailed unpaired t-test with Welch’s correction, t = 16.29, ****P < 0.0001 between young and old; young, n = 1,312 cells, old, n = 1,261 cells) and Neuroblast (CAS-Up: two-tailed unpaired t-test with Welch’s correction, t = 6.254, ****P < 0.0001 between young and old; CAS-Down: two-tailed unpaired t-test with Welch’s correction, t = 5.812, ****P < 0.0001 between young and old; young, n = 859 cells, old, n = 146 cells) in Dulken 2019 dataset. Box plots depict the median and interquartile range, with whiskers indicating minimum and maximum values. **i**, Schematic illustration of GLM prediction model. **j**, Assessment of the ability of the GLM prediction model to selected cell types in dentate gyrus. Size of dots corresponds to percentage of Precision score in terms of their actual age. Color of dots corresponds to their actual age. CAS, core aging signature, C-R, Cajal-Retzius, DEG, differentially expressed gene, GC, granule cell, GLM, generalized linear model, IPC, intermediate progenitor cell, NB/IMN, neuroblast and immature neuron, NS, not significant, OPC, oligodendrocyte precursor, qNSC, quiescent neural stem cell, SMC, smooth muscle cell and ST, spatial transcriptomics. Source data

Next, we aimed to identify gene sets reporting age-dependent molecular changes of the entire niche. To this end, we employed two approaches to quantify differential gene expression profiles: pairwise comparisons (Extended Data Fig. 7) and real-time trajectories (Fig. 5). We first performed pairwise comparisons for each cell type between each age to calculate DEGs (Extended Data Fig. 7a). In agreement with previous studies^66^, the number of DEGs between the neighboring timepoints was smaller than the number of DEGs across multiple timepoints (that is, young versus old) (Extended Data Fig. 7b), suggesting that gradual changes develop into significance across multiple timepoints. We observed similar results when we performed pairwise comparisons for the entire DG in the ST dataset (Extended Data Fig. 7c,d). Nevertheless, classical pairwise comparisons are inherently limited by several factors, such as number of cells sequenced and number of genes detected, that largely influence obtained *P* values (Extended Data Fig. 7a). Furthermore, pairwise comparisons are not able to fully capture dynamic changes of gene expression patterns over multiple timepoints^66^.

Thus, we took an alternative approach to create gene expression signatures using real-time ordering^67,68^. We first calculated genes ordered in temporal sequence (that is, young to middle-age to old) of the entire DG derived from the ST data. We then chose the genes in temporal order from the main neurogenic niche populations that overlapped with the entire DG and were shared by at least two niche populations (Fig. 5a and Methods). We curated one core gene set of 95 genes for the upregulation signature (hereafter referred to as Core Aging Signature (CAS)-Up signature) and another core gene set of 248 genes for the downregulation signature (hereafter referred to as CAS-Down signature) (Fig. 5b). These genes represented a minimal number of genes showing the same trend across multiple cell types and within the entire DG (1.5% and 3.6% of all regulated genes). Gene set enrichment analysis (GSEA) revealed that top Gene Ontology (GO) terms enriched with the CAS-Up signature were related to various inflammatory responses, whereas the top GO terms enriched with the CAS-Down signature were related to neuronal physiology (Fig. 5c). CAS-Up and CAS-Down were able to detect age-dependent changes already in middle-aged mice (Fig. 5d–h). Indeed, we cross-validated our CASs using a published dataset of aging hippocampus and SVZ of the lateral ventricle^27,69^ (Extended Data Fig. 7e–h). We further confirmed the robustness of the CAS by using a generalized linear model (GLM)-based machine learning strategy and found that, in most cell types, the predicted cellular age was consistent with their original age (Extended Data Fig. 7i,j and Methods). Of note, some cell types could be more robustly classified than others: for example, qNSC has relatively non-specific prediction of the old age, suggesting an early transcriptomic alteration that made it challenging to separate middle-age from old age. This finding is in agreement with gene expression analyses within the population of qNSCs, showing that qNSCs already undergo substantial molecular changes at middle-age (Fig. 3), and also corroborates previous data suggesting that qNSCs undergo relatively early molecular aging^22^.

### Regional inflammation is a hallmark of DG aging

**Fig. 6:**
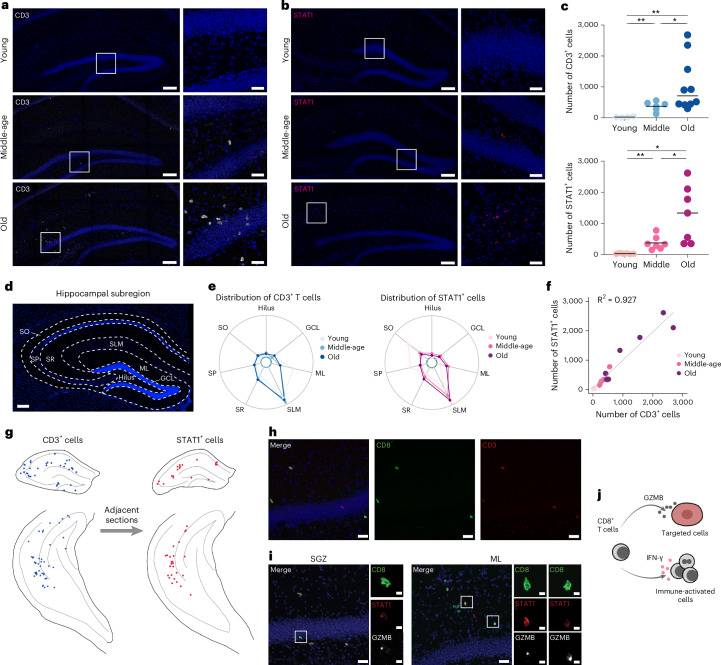
Age-dependent accumulation of T cells in the mouse hippocampus. **a**, Representative images showing age-dependent accumulation of T cells in the mouse hippocampus. **b**, Representative images showing age-dependent accumulation of IFN-γ-responding (STAT1^+^) cells in the mouse hippocampus. **c**, Quantitation of T cells (upper) in hippocampus among different ages (young: 20 ± 9 cells; middle-age: 374 ± 63 cells; old: 1,055 ± 271 cells; two-tailed unpaired *t*-test with Welch’s correction, **P = 0.0023 between young and middle-age, *P = 0.0346 between middle-age and old, **P = 0.0042 between young and old) and STAT1^+^ cells (lower) in hippocampus among different ages (young: 28 ± 6 cells; middle-age: 373 ± 82 cells; old: 1,297 ± 344 cells; two-tailed unpaired *t*-test with Welch’s correction, **P = 0.0054 between young and middle-age, *P = 0.0363 between middle-age and old, *P = 0.0102 between young and old). **d**, Illustration of hippocampal subregions. **e**, Radar plots showing the spatial distribution of T cells (left) (young: hilus 5.0%, GCL 5.0%, ML 27.5%, SLM 41.3%, SR 11.3%, SP 7.5%, SO 2.4%; middle-age: hilus 5.6%, GCL 3.2%, ML 11.5%, SLM 47.2%, SR 16.3%, SP 8.5%, SO 7.7%; old: hilus 4.5%, GCL 6.9%, ML 11.5%, SLM 43.9%, SR 17.4%, SP 8.9%, SO 6.9%) and STAT1^+^ cells (right) (young: hilus 0.0%, GCL 7.3%, ML 4.2%, SLM 28.1%, SR 9.4%, SP 20.8%, SO 30.2%; middle-age: hilus 5.4%, GCL 7.8%, ML 14.8%, SLM 37.9%, SR 13.2%, SP 7.8%, SO 13.1%; old: hilus 3.0%, GCL 5.7%, ML 12.2%, SLM 43.9%, SR 16.1%, SP 8.8%, SO 10.3%) within hippocampal subregions. **f**, Correlation between the number of T cells and STAT1^+^ cells in the old hippocampus. **g**, Rendered images showing correlated spatial distribution of T cells (blue dots) and STAT1^+^ cells (red dots) in the old mouse hippocampus. **h**, Representative images showing that most T cells in the old mouse DG were CD8^+^ (*n* = 10 mice). **i**, Representative images showing the inflammatory and cytotoxic phenotypres of CD8^+^ T cells in the old mouse DG (*n* = 4 mice). **j**, Schematic illustration of the pro-inflammatory and cytotoxic phenotypes of T cells in the old mouse hippocampus. GCL, granule cell layer; ML, molecular layer; SLM, stratum lacunosum-moleculare; SO, stratum oriens; SP, stratum pyramidale; SR, stratum radiatum. All data are presented as mean ± s.e.m. Scale bars, 100 μm (20 μm zoom-in panels) (**a**,**b**,**d**) and 20 μm (5 μm zoom-in panels) (**h**–**l**). Source data

**Extended Data Fig. 8:**
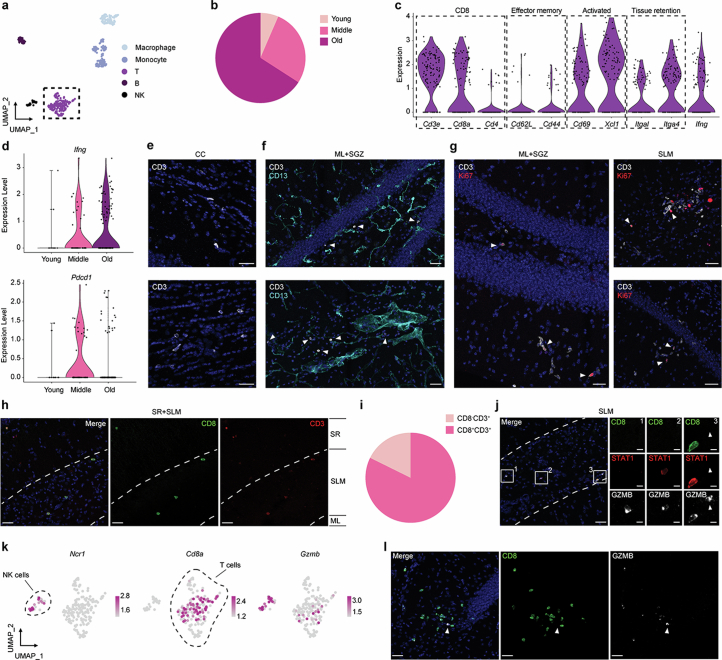
Phenotypes of age-dependent accumulative T cells in hippocampus with advancing age. **a**, UMAP plot of immune population in the current dataset. **b**, Pie chart of the proportion of T cells detected in all three ages (young: 6.5%; middle-age: 27.6%; old: 65.9%). **c**, T cells in old brains express Cd8 and phenotypes of effector memory (*Cd62L*^−^*Cd44*^*+*^), tissue retention (*Itgal*^*+*^*Itga4*^*+*^) and activation (*Cd69*^*+*^*Xcl1*^*+*^). **d**, Violin plots showing upregulated expression of IFN-γ and Pdcd1 in T cells at the advancing ages. **e**, Representative images showing the emergency of T cells in the white matter of old brains (upper: corpus callosum; lower: fimbria) (n = 10 mice). **f-g**, Representative images showing parenchyma-residential status (non-vessel-associated) (**f**) and local amplification (Ki67^+^) (**g**) of T cells in old hippocampi (n = 10 mice). **h**, Representative images showing the majority of T cells in the non-DG hippocampal subregions of old brains were CD8^+^. **i**, Pie chart of the proportion of CD8^+^ T cells in all CD3^+^ T cells of the old mouse brain (CD8^+^CD3^+^ T cells: 82.3%; CD8^−^CD3^+^ T cells: 17.7%; n = 10 mice). **j**, Representative images showing the inflammatory and cytotoxic phenotypres of CD8^+^ T cells in the old mouse non-DG jippocampus (n = 4 mice). **k**, Feature plots of NK cell-specific gene (*Ncr1*), CD8^+^ T cell-specific gene (*Cd8a*) and cytotoxicity-related gene (*Gzmb*). Color gradient indicates the log-normalized gene expression level. **l**, Representative images of GZMB^+^CD8^−^ cells in the old DG (n = 4 mice). GCL, granule cell layer, ML, molecular layer, NK, natural killer, SLM, stratum lacunosum moleculare, SO, stratum oriens, SP, stratum pyramidale and SR, stratum radiatum. All data were presented as mean ± SEM. Scale bars, (**a-b**) 100 μm (20 μm zoom-in panels), (**d**) 200 μm and (**h-i**) 20 μm (5 μm zoom-in panels).

Top enriched GO terms of CAS-Up were related to T-cell-mediated inflammation (Fig. 5c). Even though the transcriptomic analyses had been limited to cells that are unambiguously of DG origin, we detected several immune populations, including T cells, that may have infiltrated brain parenchyma (Extended Data Figs. 1a and 8a). Thus, we further analyzed their transcriptional profile and found that T cells, mainly detected in the aged DG (Extended Data Fig. 8b), were mostly CD8^+^, expressing markers of the effector memory phenotype (*Cd62L*^−^*Cd44*^+^) and showed high expression levels of genes associated with tissue retention (*Itga4* and *Itgal*) and activation (*Cd69* and *Xcl*). These findings are fully in line with recent studies showing age-dependent accumulation of T cells in multiple brain regions, including the SVZ^27,70,71^ (Extended Data Fig. 8c). Notably, T cells in aged mice showed upregulation of *IFN-γ* and *Pdcd1*, genes that are nearly absent in aged T cells of the peripheral blood^27^, indicative of brain resident, inflammatory T cells (Extended Data Fig. 8d). Using immunofluorescent staining, we confirmed the emergence of T cells and STAT1^+^ cells that respond to their key cytokine, IFN-γ, in the aged mouse hippocampus (Fig. 6a–g and Extended Data Fig. 8e–g). Notably, accumulation of T cells was not unique to the DG but, rather, detected across the entire hippocampus and white matter tracts. Although most of the age-dependent T cells in the old brain are CD8^+^ and STAT1^+^, a few of them are GZMB^+^ that display a cytotoxic phenotype (Fig. 6h,i and Extended Data Fig. 8h–j). We also noticed there are some GZMB^+^CD8^−^ cells, presumably natural killer (NK) cells, as described previously (Extended Data Fig. 8k,l)^72^.

**Fig. 7:**
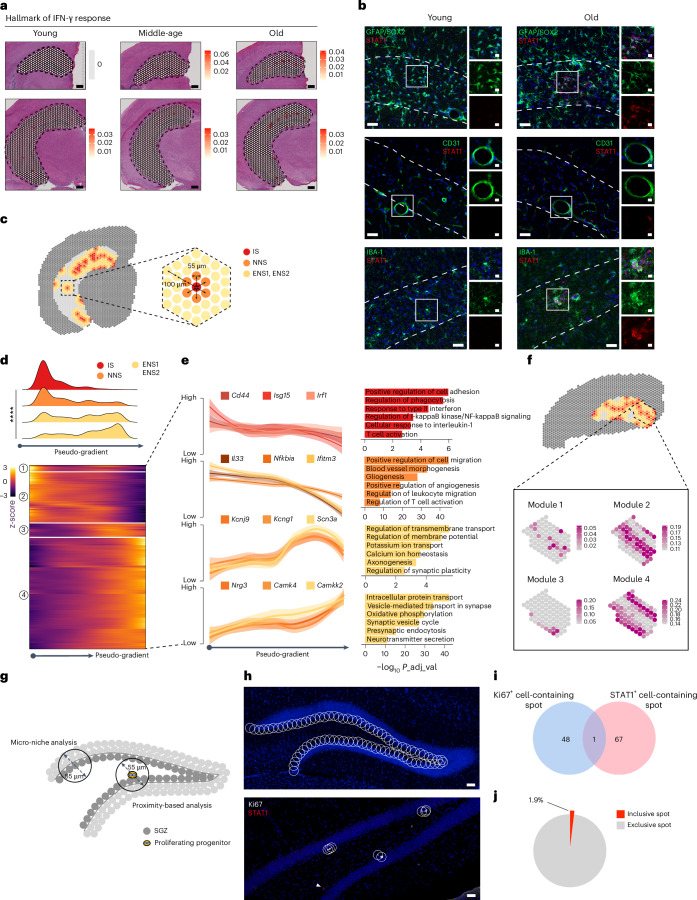
T-cell-associated inflammation with advancing ages. **a**, Spatial feature plots of the hallmark of IFN-γ response showing age-dependent increase in DG. Color gradient indicates the log-normalized gene expression level. **b**, Representative images showing age-dependent IFN-γ-responding (STAT1^+^) astrocyte (upper), endothelial cell (middle) and microglia (lower) in the marginal zone between the DG and the CA (*n* = 4 mice for each condition). **c**, Schematic illustration of the distance-based spatial hierarchy of Visium spots. **d**, Pseudo-spatial alignment of different types of spots showing differential distribution along the pseudo-spatial axis (upper; one-way ANOVA, ****P < 0.0001) and dynamic expression patterns (lower). Numbers in circle indicate gene modules. Color gradient indicates the z-score of gene expression. **e**, Pseudo-spatial gene expression trajectories showing different expression patterns of different types of spots in the old mouse hippocampus. All GO terms are shown by an adjusted *P* < 0.05 with Benjamini–Hochberg correction. Shading indicates 95% confidence interval. **f**, Spatial visualization of all four pseudo-spatial modules shows an inside-out ordering. Color gradient indicates the expression level of individual module score. **g**, Schematic illustration of microniche analysis and proximity analysis. **h**, Representative images illustrating microniche analysis (upper) and proximity analysis (lower). **i**, Venn diagram indicating the overlap between the Ki67^+^ cell-containing spot and the STAT1^+^ cell-containing spot. **j**, Pie chart of the percentage of inclusive spots that contained both Ki67^+^ cells and STAT1^+^ cells. All data are presented as mean ± s.e.m. Scale bars, 100 μm (20 μm in zoom-in panels). Source data

**Extended Data Fig. 9:**
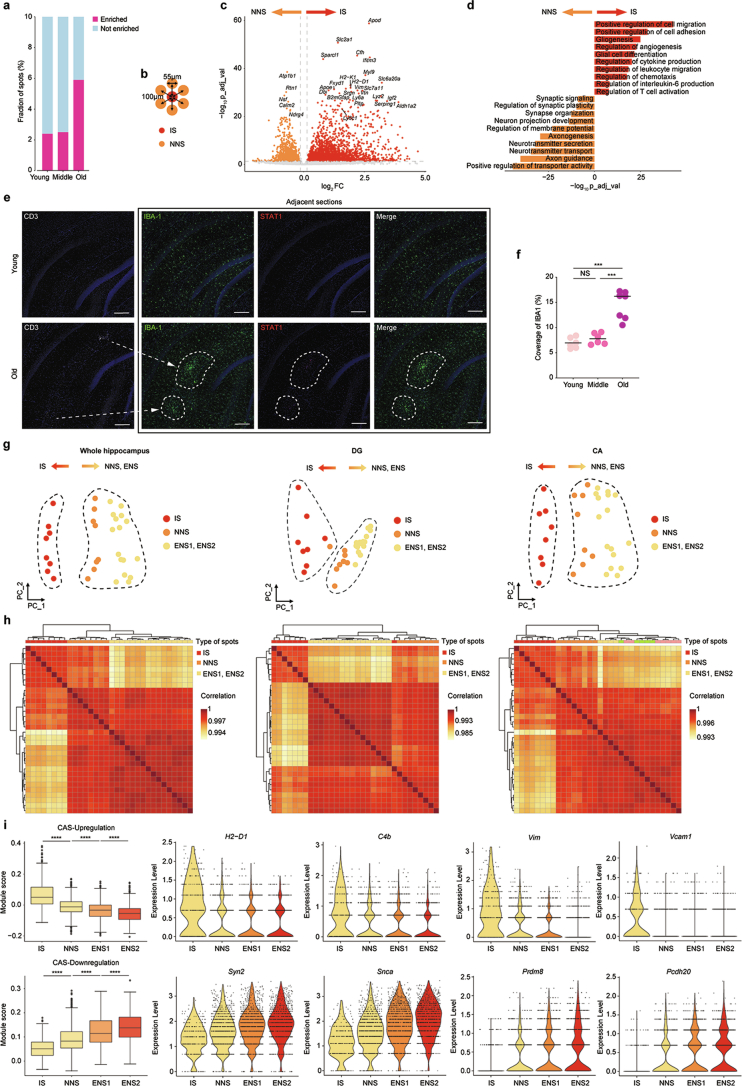
Neuroinflammatory signature in hippocampus with advancing age. **a**, The proportion of spots enriched with IFN-γ signature is increased in the old hippocampus (young: 2.4%; middle-age: 2.5%; old: 5.9%). **b**, Pictogram illustration of inflammatory spots (IS) and their nearest neighbor spots (NNS). **c**, Volcano plot showing the differentially expressed genes between IS and their NNS. For all dots shown in color by an adjusted *P* < 0.05, DESeq2 differential test. **d**, Gene Ontology terms enriched in IS and their NNS. All GO terms shown by an adjusted *P* < 0.05 with Benjamini–Hochberg correction. **e**, Representative images showing the spatial correlation between T cells, IFN-γ signature (STAT1^+^ cells) and reactive microglia. **f**, Quantitation of the coverage of IBA-1 signal in hippocampus between young (3 MO), middle-age (10 MO) and old (18MO) animals (young: 7.0 ± 0.4%; middle-age: 7.8 ± 0.4%; old: 14.5 ± 1.1%; two-tailed unpaired t-test with Welch’s correction, NS, *P* = 0.2103 between young and middle-age, ***P = 0.0004 between middle-age and old, ***P = 0.0002 between young and old). **g**, PCA plot of *in silico* aggregated Visium spots of (left) the whole hippocampus, DG and (right) CA in the old mouse brain. **h**, Hierarchical clustering of *in silico* aggregated Visium spots showing distinct transcriptional structure among all three types of spots in (left) the whole hippocampus, DG and (right) CA of the old mouse brain. **i**, Module scores of CAS and expression of selected genes among all three types of spots. Upper: (most left) CAS-UP score (two-tailed unpaired t-test with Welch’s correction; t = 14.99, ****P < 0.0001 between IS and NNS, t = 10.35, ****P < 0.0001 between NNS and ENS1, t = 8.256, ****P < 0.0001 between ENS1 and ENS2; IS, n = 299 spot, NNS, n = 967 spots, ENS1, n = 1163 spots, ENS2, n = 1005 spots) and (the rest) *H2-D1*, *C4b*, *Vim* and *Vcam1* among all three types of spots. Lower: (most left) CAS-DOWN score (two-tailed unpaired t-test with Welch’s correction; t = 12.29, ****P < 0.0001 between IS and NNS, t = 12.62, ****P < 0.0001 between NNS and ENS1, t = 7.295, ****P < 0.0001 between ENS1 and ENS2; IS, n = 299 spot, NNS, n = 967 spots, ENS1, n = 1163 spots, ENS2, n = 1005 spots) and (the rest) *Syn2*, *Snca*, *Prdm3* and *Pcdh20* among all three types of spots. Box plots depict the median and interquartile range, with whiskers indicating minimum and maximum values. Scale bars, 100 μm. Source data

As T cells are the major source of IFN-γ, we next examined gene expression patterns directly on spatially profiled tissues and identified an age-dependent increase of spots enriched with an IFN-γ response signature (hereafter referred to as inflammatory spots (ISs)) in the aged mouse hippocampus (Fig. 7a, Extended Data Fig. 9a and Methods). ISs displayed distinct transcriptional profiles compared to their nearest neighbor spots (NNSs) with upregulation of various inflammatory pathways (Extended Data Fig. 9b–d). We confirmed the emergence of ISs by immunohistochemical detection of STAT1 expression in several resident populations, including astrocytes, endothelial cells and microglia, in old age (Fig. 7b). Furthermore, we detected a spatial correlation of T cells, STAT1^+^ inflammatory cells and reactive microglia displaying activated morphology, suggesting a putative causal relationship between T cell accumulation and inflammation in the aged hippocampus (Extended Data Fig. 9e,f).

Notably, inflammatory signatures were not evenly distributed in the mouse hippocampus but, rather, peaked around the stratum lacunosum moleculare (SLM), the marginal zone between the CA and the DG (Fig. 6e). Thus, we characterized the spatial molecular architecture of the pro-inflammatory microenvironment in the old mouse brain by dissecting the ISs, NNSs and two additional layers of extended neighbor spots (ENSs) (hereafter referred to as ENS1 and ENS2) (Fig. 7c). First, we examined the global transcriptional structure and found that, although all three types of spots formed their own clusters, the ISs segregated from the NNSs and the ENSs despite their location in hippocampal subregions, indicating their distinct transcriptomes and bona fide inflammatory nature (Extended Data Fig. 9g,h). The transcriptional profile of inflammatory gradients also showed correlation with the CASs, suggesting that ISs show enhanced aging features compared to neighboring areas (Extended Data Fig. 9i).

We further constructed a continuous expression trajectory ordered from IS to NNS to ENS, with all three types of spots showing differential distribution along the pseudotime axis (Fig. 7d). Gene expression patterns along the pseudotime trajectory were sequentially expressed from IS to ENS, with modules 1 and 2 enriched for genes related to inflammatory response, gliogenesis and vasculature remodeling and modules 3 and 4 enriched for genes related to ion channel homeostasis and neurotransmitter transport (Fig. 7e). Furthermore, these modules showed an obvious spatial distribution, ordered in an inside-out pattern from the IS to adjacent regions (Fig. 7f). We performed microniche analysis by tiling the DG into spots with a 55 µm radius to measure the co-occurrence of STAT1^+^ cells and proliferating progenitors^33^ (Fig. 7g,h). These analyses showed that Ki67^+^ progenitors and STAT1^+^ inflammatory cells are largely exclusive to each other (Fig. 7i,j). Thus, our results identified an inflammation-associated gene expression gradient, suggesting that age-dependent accumulation of T cells in the marginal zone of the DG may represent an initial step of age-dependent neuroinflammation that spreads to adjacent regions, causing eventually age-related impairment of hippocampal plasticity.

### Early onset of inflammation decreases neurogenic activity

**Fig. 8:**
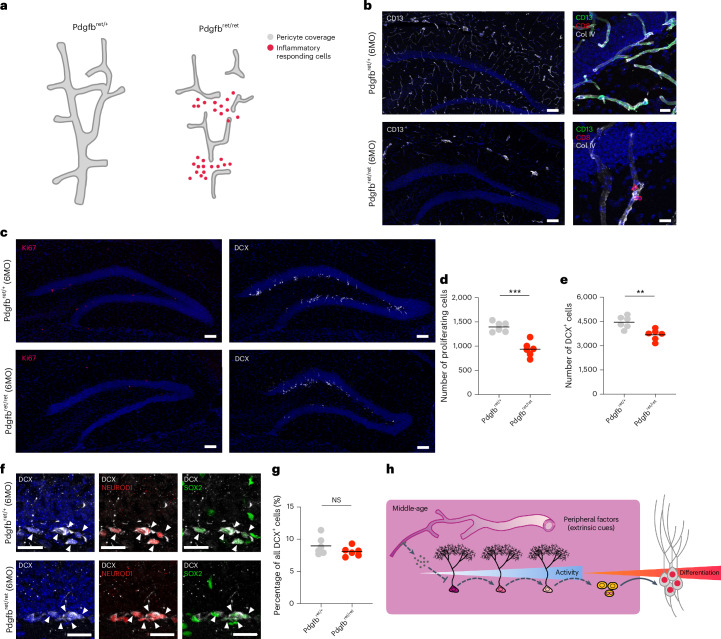
Loss of immune privilege leads to reduced neurogenic activity in the BBB-deficient mouse brain. **a**, Schematic illustration of the pericyte-deficient and inflammatory phenotypes of the Pdgfb^ret/ret^ mouse model. **b**, Immunofluorescent staining of pericyte marker (CD13) showing deficient pericyte network and infiltrated CD8^+^ T cells in the Pdgfb^ret/ret^ mouse hippocampus compared to the control (Pdgfb^ret/+^) animals. **c**, Representative images showing reduced proliferating cells (Ki67^+^) (left) and reduced newly born neuronal cells (DCX^+^) (right) in the Pdgfb^ret/ret^ mouse SGZ. **d**, Quantitation of Ki67^+^ cells in the SGZ between Pdgfb^ret/+^ and Pdgfb^ret/ret^ (Pdgfb^ret/+^: 1,396 ± 42 cells, *n* = 6 mice; Pdgfb^ret/ret^: 937 ± 64 cells, *n* = 6 mice; two-tailed unpaired *t*-test with Welch’s correction, ***P = 0.0002). **e**, Quantitation of DCX^+^ cells in the SGZ between Pdgfb^ret/+^ and Pdgfb^ret/ret^ (Pdgfb^ret/+^: 4,457 ± 155 cells, *n* = 6 mice; Pdgfb^ret/ret^: 3,673 ± 137 cells, *n* = 6 mice; two-tailed unpaired *t*-test with Welch’s correction, **P = 0.0037). **f**, Representative images showing the existence of SOX2^+^NEUROD1^+^ neuroblasts in both Pdgfb^ret/+^ and Pdgfb^ret/ret^ mouse SGZ. **g**, Quantitation of the percentage of SOX2^+^NEUROD1^+^ neuroblasts among all neuroblasts in the SGZ between Pdgfb^ret/+^ and Pdgfb^ret/ret^ (Pdgfb^ret/+^: 9.0 ± 0.6%, *n* = 6 mice; Pdgfb^ret/ret^: 8.1 ± 0.3%, *n* = 6 mice; two-tailed unpaired *t*-test with Welch’s correction, NS *P* = 0.2057). **h**, Schematic illustration of the interaction between environmental factors and neurogenic activity. Whereas proliferation and generation of DCX^+^ cells are impaired in Pdgfb^ret/ret^ mice (dashed line), delayed maturation of neuroblasts is not affected in Pdgfb^ret/ret^ mice. All data are presented as mean ± s.e.m. Scale bars, 100 μm (**a**,**c**) and 20 μm (**e**). MO, months old; NS, not significant. Source data

**Extended Data Fig. 10:**
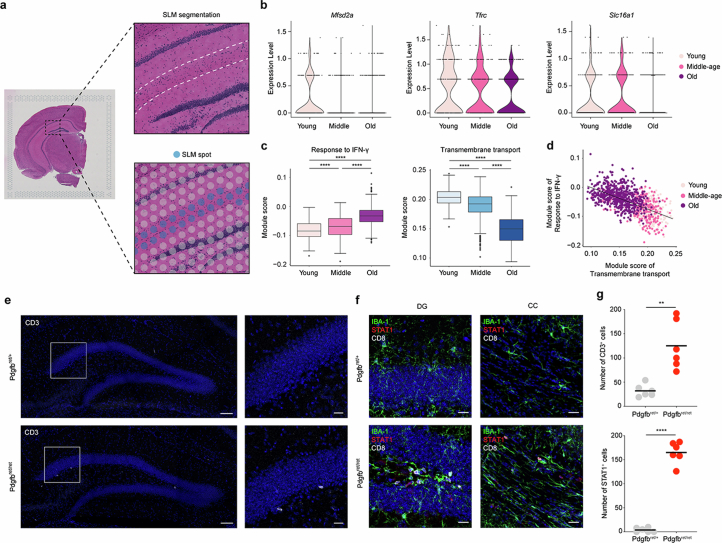
Characterization of phenotypes of Pdgfb^ret/ret^ mouse model. **a**, Schematic illustration of the manual selection of SLM spots. **b**, Violin plots showing age-dependent changes in the expression of BBB-specific (*Mfsd2a*, *Tfrc* and *Slc16a1*) genes in SLM spots. **c**, Module scores of the hallmark of IFN-γ response (two-tailed unpaired t-test with Welch’s correction; t = 4.167, ****P < 0.0001 between young and middle-age, t = 13.85, ****P < 0.0001 between middle-age and old, t = 21.38, ****P < 0.0001 between young and old; young, n = 271 spots, middle-age, n = 274 spots, old, n = 571 spots) and GO term of transmembrane transport (two-tailed unpaired t-test with Welch’s correction; t = 8.785, ****P < 0.0001 between young and middle-age, t = 19.39, ****P < 0.0001 between middle-age and old, t = 39.50, ****P < 0.0001 between young and old; young, n = 271 spots, middle-age, n = 274 spots, old, n = 571 spots) among all three ages in SLM spots. **d**, Correlation between the module scores of the hallmark of IFN-γ response and GO term of transmembrane transport in SLM spots. **e**, Representative images showing infiltration of T cells (CD3^+^ cells) in the Pdgfb^ret/ret^ mouse hippocampus. **f**, Representative images showing emergence of CD8^+^ T cells, IFN-γ signature (STAT1^+^ cells) and co-localization of reactive microglia in (left) DG and (right) CC of (lower) Pdgfb^ret/ret^ mouse compared to (upper) control (Pdgfb^ret/+^) animals. **g**, Quantitation of the number of (upper) CD3^+^ T cells (Pdgfb^ret/+^: 32 ± 5 cells; Pdgfb^ret/ret^: 125 ± 20 cells; two-tailed unpaired t-test with Welch’s correction, ** *P* = 0.0051 between Pdgfb^ret/+^and Pdgfb^ret/ret^) and (lower) STAT1^+^ cells in DG between 6 MO Pdgfb^ret/+^ and Pdgfb^ret/ret^ animals (Pdgfb^ret/+^: 4 ± 2 cells; Pdgfb^ret/ret^: 165 ± 9 cells; two-tailed unpaired t-test with Welch’s correction, **** *P* < 0.0001 between Pdgfb^ret/+^and Pdgfb^ret/ret^). BBB, blood-brain barrier, CC, corpus callosum, DG, dentate gyrus and SLM, stratum lacunosum moleculare. Scale bars, (**e**) 100 μm (20 μm in zoom-in panels) and (**f**) 20 μm. Source data

As characterized above, the age-dependent accumulation of T cells mainly peaked at the SLM, the embryonic homology to the brain border that also displayed enhanced aging features (Fig. 6e and Extended Data Fig. 9h). We wondered whether the SLM-related bordering function may be impaired during aging. To this end, we subsetted Visium spots located at the SLM and identified downregulation of several BBB-specific genes—that is, *Mfsd2a*, *Tfrc* and *Slc16a1* (Extended Data Fig. 10a,b). This transcriptional change is associated with a reciprocal correlation between the response to IFN-γ signaling and BBB-related function (Extended Data Fig. 10c,d). To directly examine how inflammation affects neurogenic activity, we used the Pdgfb^ret/ret^ mouse model that shows BBB deficiency due to the loss of pericytes and that develops early-onset neuroinflammation (Fig. 8a)^73–75^. We first confirmed the previously described phenotype of Pdgfb^ret/ret^ mice, showing a decrease in pericyte coverage that was associated with the infiltration of T cells, especially CD8^+^ T cells, and increased inflammatory response, as measured by STAT1 expression, and the presence of reactive microglia labeled with IBA-1 in the hippocampus (gray matter) and the corpus callosum (white matter) in middle-aged mice (Fig. 8b and Extended Data Fig. 10e–g). Next, we measured the number of proliferative progenitors and newborn, DCX-labeled neurons in the DG. Strikingly, we observed a significant decrease in the numbers of proliferative progenitors and newborn neurons in the DG of Pdgfb^ret/ret^ mice, indicating impaired neurogenesis in the context of a dysregulated immune microenvironment (Fig. 8c–e). However, the fraction of SOX2^+^ neuroblasts, which we had identified to be increased with advancing age (Extended Data Fig. 5d–f), remained constant between mutant and control animals, suggesting that the maturation of early neuronal progeny may be, at least partially, regulated by additional age-dependent mechanisms (Fig. 8f–h). Thus, our findings provide insights into the functional relevance of age-dependent inflammation in the regulation of hippocampal neurogenesis and show that early-onset inflammation in the DG is sufficient to impair neurogenesis.

## Discussion

Using scRNA-seq combined with spatially resolved transcriptomics, we characterized age-associated molecular changes in the mouse DG across the entire adult lifespan. Single-cell technologies allowed for insights in the mechanisms of aging in a plethora of tissues and in the context of disease^66,76,77^. Our data provide a powerful resource to reveal the mechanisms that are linked with aging in the mammalian hippocampus. First, our findings reveal the lifelong molecular changes of the entire neurogenic lineages that conceptually extends current knowledge: age-dependent changes of neurogenesis start not only as early as middle-age but also last throughout the entire adult lifespan. Beyond the activation of qNSCs, neurogenic aging also affects the differentiation of fate-committed neural progeny. Early entry to quiescence was suggested as a protective approach to avoid premature depletion of the stem cell pool^78,79^. The data presented here suggest that a continuous deepening of NSC quiescence, together with delayed maturation of neuronal progeny, may eventually cause impaired neurogenesis in later stages of life. We focused and extensively analyzed age-dependent molecular alterations within the neurogenic lineage. However, the scRNA-seq and ST data provided will allow for future in-depth transcriptomic analyses of a plethora of brain cell types across the adult lifespan.

Several recent studies used single-nuclei RNA sequencing (snRNA-seq) to derive molecular maps of the human and non-human primate DG across the lifespan^21,25,26,80,81^. Despite the possibility to use snRNA-seq of human tissues, mechanistic attempts to causally link gene expression with functional outcomes will largely rely on rodent models of aging. Thus, the mouse transcriptomic dataset of the aging DG provided here will be a valuable resource to the field and a fundamentally important addition to existing human and primate datasets. Furthermore, whole-cell scRNA-seq versus snRNA-seq may have its respective advantages: within the dataset presented here, we could, for example, clearly distinguish qNSCs from parenchymal astrocytes, a distinction that is apparently critical to identify age-related changes within the neurogenic lineage. However, clear classification of qNSCs versus astrocytes appeared to be highly challenging or impossible in previously obtained datasets using single-nuclei approaches of mouse or human tissues^25,26,82^. The dataset provided here represents a transcriptomic reference that may eventually allow for the identification of bona fide neural stem cells apart from astroglia combined with advanced computational approaches—for example, refs. ^83,84^—that may be of particular importance to address the remaining uncertainties regarding adult hippocampal neurogenesis in humans^85^.

Seminal work previously identified neuroinflammation as a key aging signature of the other main neurogenic niche in the mouse brain, the SVZ^27^. Indeed, it was found that T cells invade the SVZ and appear to clonally expand within the brain parenchyma. Together with other work, these previous data strongly support the idea that aging within the brain is associated with inflammation, which has been coined as inflammaging^27,86^. Our data recapitulate findings derived from the SVZ and show that aging causes global changes in the transcriptome of the entire DG that are associated with T-cell-mediated inflammatory responses, which we confirmed by detecting increased T cell numbers within the DG. Notably, spatially resolved transcriptomics allowed for the identification of the regional emergence of inflammatory hotspots in the marginal zone. Given that the marginal zone in the hippocampus shows embryonic homology to the brain border—that is, meninges—which show an age-dependent accumulation of T cells, it may represent a target to attenuate age-dependent alterations of hippocampal neurogenesis. Future work, guided by the molecular signatures at inflammatory hotspots and their surrounding tissues, will aim to identify the cause and regulatory cascades that mediate the invasion and consequences of T-cell-mediated inflammation in the aging hippocampus. Notably, we show here that invasion of inflammatory T cells is not just a bystander effect but that experimental enhancement of immune cell invasion causes reduced neurogenesis. However, premature invasion of immune cells did not cause a complete recapitulation of the effects of aging on neurogenesis, as the delayed maturation and extended expression of progenitor markers (such as SOX2) was not affected by Pdgfb deletion. Thus, future work will aim to dissect the exact contributions of distinct mechanisms causing molecular consequences of age within the DG circuit. The single-cell and spatially resolved transcriptomics data provided here represent a starting point to the field to reveal the molecular consequences that are associated with advancing age in the mammalian hippocampus.

## Methods

### Experimental animals

All animal experiments were approved by the Cantonal Commission for Animal Experimentation of the Canton of Zurich, Switzerland (ZH190/19 and ZH126/20), in accordance with national and cantonal regulations and the Stockholms Norra Djurförsöksetiska Nämnd (20785-2020), Sweden, performed in accordance with the respective guidelines. Mice were group housed in ventilated cages (21–23 °C) under a 12-h dark/light cycle with ad libitum access to food and water.

The C57BL/6JRj mice (3 MO, 9–11 MO and 16–21 MO) were obtained from Janvier France and maintained in local animal facilitates for 1 week before experiments. For scRNA-seq and ST experiments, mice at the age of 3 months, 9–11 months and 16–21 months, referred to as ‘young’, ‘middle-age’ and ‘old’, of mixed sex were used. For histological analysis, mice at the age of 3 months, 10 months and 18 months of mixed sex were used. The Gli1-Cre^ERT2^;tdTomato mouse line (Gli1-Cre^+/−^Ai14^+/−^) was generated by crossing Gli1-Cre^ERT2^ mice (Gli1^tm3(cre/ERT2)Alj^; The Jackson Laboratory, 007913) and CAG tdTomato (Ai14;B6.Cg-Gt(ROSA)^26Sortm14(CAG-tdTomato)Hze^; The Jackson Laboratory, 007914) reporter line^45,47^. At the age of 3 months, 10 months and 17 months, referred to as ‘young’, ‘middle-age’ and ‘old’, mice of mixed sex underwent the labeling of NSCs that was achieved by two consequential intraperitoneal (i.p.) injections of TAM (180 mg kg^−1^ (body weight); Sigma-Aldrich). The Nestin-EGFP (B6.Cg-Tg(Nes-EGFP)1Yamm/Rbrc) mouse line was described previously^43,47^. Mice at the age of 3 months, 10 months, 18 months and 24 months, referred to as ‘young’, ‘middle-age’, ‘old’ and ‘very old’, of mixed sex were used for experiments. The Pdgfb^ret/ret^ (Pdgfb-tm(ret)) mouse line was described previously^73–75^. Homozygous (Pdgfb^ret/ret^) and heterozygous (Pdgfb^ret/+^) female mice at the age of 6 months were used for experiments.

### Tissue processing for scRNA-seq

In total, 17 mice were used for the young (*n* = 5), middle-aged (*n* = 6) and aged (n = 6) groups (Supplementary Fig. 1). Mice were first euthanized via cervical dislocation, followed by taking out brains and then microdissection of the DG^87^. The dissected DGs from the same age were pooled and dissociated using Neural Tissue Dissociation Kit (P) (Miltenyi Biotec) and further cleaned using Myelin Removal Beads II (Miltenyi Biotec) according to the manufacturer’s instructions. In brief, pooled tissue was enzymatically digested for 35 min at 37 °C, followed by manual trituration with fire-polished pipette tips and filtered with 40-μm strainers. Cell suspension was incubated with Myelin Removal Beads for 15 min on ice, followed by cleaning with passing through a magnet LS column (Miltenyi Biotec). Single-cell suspension was sorted from debris with the target number of 50,000 in an influx cell sorter using a 130-μm nozzle (BD FACSAria II).

### Tissue processing for ST

In total, 12 mice were used for the young (*n* = 4), middle-aged (*n* = 4) and aged (*n* = 4) groups (Supplementary Fig. 2). Mice were first anesthetized via i.p. injection of a lethal dose of pentobarbital and then transcardially perfused with warm HBSS (CaCl_2_^−^, MgCl_2_^−^, HEPES 10 mM and D-glucose 5 mM), followed by taking out brains. Brains were embedded in OCT (Tissue-Tek) and snap frozen at −50 °C to −60 °C in a bath of isopentane and dry ice and stored at −80 °C until use. Two to three coronal sections per brain were cyrosectioned, referring to anterior and posterior hippocampus at a thickness of 10 μm onto the Visium Spatial Gene Expression Slide. For anterior sections, we fitted two into one sequencing area. For posterior sections, we fitted one into one sequencing area. All sections had an RNA integrity number (RIN) greater than 8, measured in a Bioanalyzer (Agilent). In total, 16 sequencing areas with 24 brain sections were profiled (Supplementary Fig. 2).

### Library preparation and sequencing for scRNA-seq and ST

Single-cell cDNA libraries were constructed using a Chromium Single Cell 3′ Reagent Kit (version 3.1; 10x Genomics). In brief, 20,000 cells were loaded for each library with the aim to recover 10,000 cells. All downstream steps of library construction followed the manufacturer’s instructions. Spatial cDNA libraries were constructed using a Visium Spatial Gene Expression Kit (10x Genomics). Permeabilization time was determined as 18 min using a Visium Spatial Tissue Optimization Slide & Reagent Kit (10x Genomics). All downstream steps of library construction followed the manufacturer’s instructions. All reagents used in this study are detailed in Supplementary Table 1.

Single-cell and spatial cDNA libraries were pooled and indexed using a Dual Index Kit (10x Genomics), fitting into one batch, and sequenced on an Illumina NovaSeq 6000 according to 10x Genomics recommendations. Raw data of sequencing were analyzed using Cell Ranger (version 6.0.2) for scRNA-seq and Space Ranger (version 1.2.0) for ST.

### scRNA-seq data analyses

Output of Cell Ranger was processed using Seurat (version 4.3) package. Low-quality cells were filtered out (genes detected < 800, mitochondrial genes > 20%). Standard data processing workflow of Seurat was performed for each timepoint using ‘NormalizeData’ (scale.factor = 10,000), ‘FindVariableFeatures’ (nfeatures = 5,000), ‘RunPCA’ (npcs = 50), ‘RunUMAP’ (number of principal components (PCs) between 10 and 30), ‘RunTSNE’ (number of PCs between 10 and 30), ‘FindNeighbors’ (number of PCs between 10 and 30, decided by running ‘ElbowPlot’) and ‘FindClusters’ (resolution = 1.0) functions. We performed DoubletFinder (version 2.0.3) to remove potential doublets before we merged all libraries. Estimated doublet rate was adjusted according to 10x Genomics.

### ST data analyses

Output of Space Ranger was processed using Seurat (version 4.3) package and Cloupe software (Loupe Browser version 6; 10x Genomics). Spots with technical artifacts (for example, folding and cryo-damage) were removed on Cloupe. Standard data processing workflow of Seurat was first performed for each section using ‘SCTransform’ (variable.feature.n = 5,000). SCTransform builds regularized negative binomial models of gene expression. It is suggested that, compared to the log normalization, SCTransform accounts for technical artifacts while preserving biological variance^88^. All libraries were then merged to perform dimensionality reduction using ‘RunPCA’, ‘RunUMAP’, ‘RunTSNE’, ‘FindNeighbors’ and ‘FindClusters’ functions.

### Cell and spot type annotation

For scRNA-seq, we first determined cluster-specific DEGs by performing the ‘FindAllMarkers’ (test.use = mast) function from Seurat. Then, we annotated cell type using cell-type-specific markers from literature (Extended Data Fig. 1a)^23,34^. For ST, regional identity was assigned by intersection between unsupervised clustering results mentioned above and hematoxylin and eosin (H&E) staining visualized on Loupe software. Manual annotation of hippocampal subregions (for example, CA1, CA2, CA3 and DG) was carried out on Loupe software using results from data integration with Yao et al.^35^ and landmarks of H&E staining as reference.

### Data integration

scRNA-seq data were integrated with a previously published dataset (Dataset C)^34^. The cell type annotation from the original study was used. Batch correction was performed using the ‘harmony’ (version 0.1.0) package^89^. ST data were integrated with a previously published dataset^35^. Only hippocampal cells from the Smart-seq version 4 experiment were selected. The cell type annotation from the original study was used. Cell type prediction was calculated for each spot using the ‘FindTransferAnchors’ and ‘TransferData’ functions in Seurat. In particular, the ‘FindTransferAnchors’ function was used to identify the anchors between scRNA-seq and ST using ‘SCT’ normalization. The ‘TransferData’ function was used to transfer the cluster identities of scRNA-seq to the Visium spots. Default parameters were used.

### Cell type mapping onto Visium spots

Cell type mapping of spatial data was performed using tangram-sc version 1.0.4 (ref. ^36^). The workflow was based on the available squidpy version tutorial. All spatial samples were loaded in one data object and filtered on coordinates to include only the ML, hilus and GC layer spots. For scRNA-seq data, the mitochondrial genes were filtered out, and the data were normalized. Tangram built-in methods were used for pre-processing, which left 1,104 marker genes as training genes. Before running the alignment, the spatial data were normalized and log1p transformed using scanpy version 1.10.1. The alignment was run in ‘cells’ mode with 1,000 epochs and rna_count_based as density prior. To visualize the annotation of cell types in space, the colormap argument ‘perc’ was set to 0.02 and spot_size to 500 to better display the selected coordinates.

### Differential gene expression and GSEA

For scRNA-seq, we performed pairwise differential gene expression to identify DEGs using the ‘FindAllMarkers’ or ‘FindMarkers’ (test.use = mast) functions from Seurat. DEGs were selected using cutoffs of adjusted *P* < 0.05 and avg_log2fc > 0.25. For ST, raw data were first pooled to generate pseudo-bulk aggregates and processed using DESeq2 to identify DEGs. DEGs were selected using a cutoff of adjusted *P* < 0.05. GSEA was performed using the Metascape database related to GO Biological Process and KEGG pathway. An overrepresentation test was carried out with a hypergeometric test of *P* < 0.05 and a minimal overlap of three genes. The list of TFs was curated from the AnimalTFDB (version 3.0) database^90^.

### Pseudotemporal trajectory analysis

We first calculated pseudotime score of individual cells or spot in terms of age or annotation (for neurogenic lineages), zonation (for vascular compartment) and inflammatory status (for inflammatory gradient in ST) and constructed gene expression trajectories using the Monocle 2 package with default parameters. Next, we ordered genes with similar expression dynamics into modules that reflect lineage progression (neurogenic lineages), functional aging (qNSC), vascular zonation (vascular populations) and inflammatory gradient (ST).

### Real temporal trajectory analysis

We clustered gene expression patterns in time series using a fuzzy c-means clustering algorithm, ‘Mfuzz’, as described previously^91^. First, we selected the top 5,000 variable genes from Seurat objects of each cell type to make temporally ordered genes comparable among different cell types and calculated their average expression value. Then, we clustered gene expression data using soft threshold (‘mestimate’ with default parameters, number of clusters = 8).

### GRN analysis

To identify GRNs and infer their activity, we performed the SCENIC (version 1.24.1) algorithm. First, we sequentially ran GENIE3 to identify co-expressed genes and RcisTarget to link TFs and their putative targets using default parameters. TFs of mm9 were used as reference. Then, we used AUCell to score activity of each GRN (regulon). Finally, the regulon activity was visualized either by UMAP or onto pseudotime trajectory.

### Random forest regression model

To predict differentiation scores for qNSCs across ages, we employed a random forest regression model from the ‘caret’ package. We first took the whole neurogenic lineages (for example, qNSC, aNSPC and NB/IMN) and identified the top DEGs between each cell types using the ‘FindAllMarkers’ function from Seurat. Cells were projected onto a pseudo-differentiation axis by fitting a principal curve over the first seven PCs. We then trained a random forest regression using the ‘train’ function (method = ‘ranger’) from ‘caret’ to predict the pseudo-differentiation score of cells using the top variable genes. The top 100 most important features from the random forest regression model were selected as an input to re-optimize the regression model that was used to predict the age dependent for qNSCs in our dataset.

### Age prediction model

We performed GLM-based age prediction to predict cells with corresponding ages using the ‘caret’ package. First, we performed the ‘FindVariableFeatures’ function to identify highly variable genes and selected the top 5,000 variable genes. Next, we split the scRNA-seq data into a training set and a testing set, where the training set consisted of 70% of cells from the least populated class, and the corresponding number of cells was taken from the more populated classes. To predict cells with corresponding ages in the testing set, we used the ‘train’ function (method = glmnet) from ‘caret’. Cross-validation was performed 10 times with 10% of the training data for the parameter tuning (lambda and alpha). Finally, the model was used to predict on the remaining 30% of data (test set) using the ‘predict’ function in ‘caret’.

### Module score calculation

For the module score of aging signatures in scRNA-seq data, we took the gene lists of ‘Neurogenic Aging Signature’ and ‘Core Aging Signature’ and computed the module score using the ‘AddModuleScore’ function with default parameters from Seurat. The complete gene lists are provided in Supplementary Table 3. For ‘S.Score’ and ‘G2M.Score’, we used the ‘CellCycleScoring’ function in Seurat to calculate the cell cycle activity of individual cells. For the module score of IFN-γ pathways in ST data, we first took the gene list of ‘Hallmark of IFN-γ response’ from the ‘msigdbr’ (version 7.4.1) package^92^ and then calculated the module score using the ‘AddModuleScore’ function with default parameters from Seurat. Spots with module score greater than 0 were assigned as ISs. The ‘STUtility’ (version 0.1.0) package was used to compute nearest neighborhood of ISs. The ‘AddModuleScore’ function calculates the average expression levels of a given gene set on single-cell level, subtracted by the aggregated expression of control feature sets. All analyzed features are binned based on averaged expression, and the control features are randomly selected from each bin.

### Tissue processing, immunostaining and confocal imaging

Mice were first anesthetized via i.p. injection of a lethal dose of pentobarbital and then transcardially perfused with warm D-PBS (CaCl_2_^−^ and MgCl_2_^−^), followed by 4% formaldehyde (Sigma-Aldrich) post-fixed overnight at 4 °C. Then, brains were transferred to 30% sucrose solution for cryoprotection before being cut at a thickness of 40 μm on a cryotome (Leica, SM2010R). Every sixth coronal section along the entire DG was used for immunostaining. For immunostaining, brain sections were first washed in PBS and blocked in the staining buffer (3% donkey serum and 0.5% Triton X-100 in PBS). Then, sections were incubated with primary antibodies against S100b (1:500, rabbit; Abcam), SOX2 (1:200, rat; eBioscience), GFAP (1:500, chicken; Aves Labs), Ki67 (1:500, rat; eBioscience), DCX (1:500, guinea pig; Millipore), NEUROD (1:250, goat; Santa Cruz Biotechnology), IBA-1 (1:500, goat; Novus Biologicals), CD3 (1:200, rabbit; Novus Biologicals), CD8a (1:200, rat; eBioscience), STAT1 (1:200, rabbit; Cell Signaling Technology), GZMB (1:100, goat; R&D Systems), Collagen IV (1:750, rabbit; Bio-Rad), CD13 (1:500, goat; Novus Biologicals), GFP (1:500, goat; Rockland Immunochemicals), tdTomato (1:500, goat; Rockland Immunochemicals) and tdTomato (1:500, goat; SICGEN) for two overnights in the staining buffer at 4 °C. After washing in PBS, sections were incubated with secondary antibodies against respective species (Alexa Fluro 488, Cy3 and Cy5) and DAPI (1 μg ml^−1^; Thermo Fisher Scientific) in the staining buffer for 2 h at room temperature. After washing, sections were mounted with Immun-Mount (Thermo Fisher Scientific) and stored at 4 °C until imaging. Images were taken on confocal laser scanning microscopes (Zeiss LSM800 using ZEN Pro software). All antibodies used in this study are detailed in Supplementary Table 1.

Every sixth coronal section along the entire DG was imaged per mouse using a ×20 objective (obtained numbers were multiplied by 6 to represent the number of all cells per DG/hippocampus). Fiji (ImageJ) was used for image analysis. For cell counting, radial NSCs were defined as GFAP^+^/SOX2^+^/S100b^−^ cells with a single radial process residing at the SGZ. Given that GFAP is cytoplasmic and SOX2 is nuclear, we combined them together in one channel. aNSPCs and neuroblasts were defined as Ki67^+^ or DCX^+^ cells residing at the SGZ, respectively. Microglia, endothelial cells, pericytes and T cells were identified as IBA-1^+^, CD31^+^, CD13^+^ or CD3^+^ cells, respectively. IFN-γ-responding cells were defined as STAT1^+^ cells. For signal coverage of IBA-1, all confocal images were taken under the same setting. Maximum z-stack projection was performed, and the IBA-1 channel was converted into binary mask using the same intensity. We manually drew hippocampus and DG using the ‘ROI’ function and measured the %Area using the ‘measure’ function. For the microniche analysis, a line of the SGZ at the bottom of the granule cell layer (GCL) was drawn manually, leaving the space of 2 cells apart from the hilus. Then, 55-μm circles were placed along this SGZ line with 50% overlap between each neighbor circle. For the proximity analysis, each Ki67^+^ proliferating progenitor in the SGZ was placed with a 55-μm circle where the Ki67^+^ proliferating progenitor was in the center.

### Single-molecule RNA fluorescence in situ hybridization

Tissue preparation was described above the same as for immunofluorescent staining. Instead of 40 μm, tissue was cut at a thickness of 20 μm. We first treated the tissue with hydrogen peroxide for 5 min at room temperature. To perform RNA in situ hybridization using RNAscope, we first performed antigen retrieval using target retrieval reagent (ACDBio) for 15 min at 85 °C. Sections were then treated with protease IV reagents (ACDBio) for 20 min at room temperature. Probes (Mm-Mfge8, 408771; Mm-Luzp2, 492551-C3; Mm-Sox11, 852061; Mm-Insm1, 430621-C2; ACDBio) were incubated at 40 °C for 2 h and revealed with RNAscope Multiplex Fluorescent v2 reagents (ACDBio) using TSA amplification. After in situ hybridization was completed, samples were processed for immunofluorescent staining and imaging as described above. All molecular probes and reagents used in this study are detailed in Supplementary Table 1.

### Statistics and reproducibility

Statistical analyses were performed in GraphPad Prism (version 9.5.1) or R (version 3.6.3). All results in graphs are presented as mean ± s.e.m. unless specified otherwise. All software used in this study is detailed in the Reporting Summary. Statistical significance was determined using two-tailed unpaired *t*-tests with Welch’s correction (between two groups) and one-way ANOVA (between multiple groups) with a cutoff of *P* < 0.05. Particular tests and statistical significance for individual comparisons in figures are detailed in Supplementary Table 5.

### Reporting summary

Further information on research design is available in the Nature Portfolio Reporting Summary linked to this article.

## Online content

Any methods, additional references, Nature Portfolio reporting summaries, source data, extended data, supplementary information, acknowledgements, peer review information; details of author contributions and competing interests; and statements of data and code availability are available at 10.1038/s41593-024-01848-4.

## Supplementary information

Supplementary Information Supplementary Figs. 1 and 2.

Reporting Summary

Supplementary Table 1 Key reagents used in the present study.

Supplementary Table 2 DEGs for each cell type.

Supplementary Table 3 Pseudotime values of the neurogenic aging trajectory and inflammatory gradient analysis.

Supplementary Table 4 GRN (SCENIC) values for the entire neurogenic lineage.

Supplementary Table 5 Statistical details for the main figures and extended data figures.

## Source data

Source Data Fig. 1 Numerical source data for indicated figure.

Source Data Fig. 2 Numerical source data for indicated figure.

Source Data Fig. 3 Numerical source data for indicated figure.

Source Data Fig. 4 Numerical source data for indicated figure.

Source Data Fig. 5 Numerical source data for indicated figure.

Source Data Fig. 6 Numerical source data for indicated figure.

Source Data Fig. 7 Numerical source data for indicated figure.

Source Data Fig. 8 Numerical source data for indicated figure.

Source Data Extended Data Fig. 1 Numerical source data for indicated extended data figure.

Source Data Extended Data Fig. 3 Numerical source data for indicated extended data figure.

Source Data Extended Data Fig. 4 Numerical source data for indicated extended data figure.

Source Data Extended Data Fig. 5 Numerical source data for indicated extended data figure.

Source Data Extended Data Fig. 6 Numerical source data for indicated extended data figure.

Source Data Extended Data Fig. 7 Numerical source data for indicated extended data figure.

Source Data Extended Data Fig. 9 Numerical source data for indicated extended data figure.

Source Data Extended Data Fig. 10 Numerical source data for indicated extended data figure.

## Data Availability

Transcriptomics data generated and analyzed in this study are available under Gene Expression Omnibus accession number GSE233363. Processed data (for example, Seurat object and tables of meta information) can be found at https://github.com/JessbergerLab/AgingNeurogenesis_Transcriptomics (ref. ^93^). Source data are provided with this paper.

## Code availability

Codes used to analyze the transcriptomics presented in this study are available at https://github.com/JessbergerLab/AgingNeurogenesis_Transcriptomics (ref. ^93^).
