# Supplementary material for: Multimodal transcriptomics reveal neurogenic aging trajectories and age-related regional inflammation in the dentate gyrus
Source: Nat Neurosci. 2025 Jan 6;28(2):415–30. doi: 10.1038/s41593-024-01848-4 (PMC11802457; doi:10.1038/s41593-024-01848-4)
Supplement: Supplementary file 1 — Supplementary Figs. 1 and 2. [file 41593_2024_1848_MOESM1_ESM.pdf]

nature neuroscience

Supplementary information https://doi.org/10.1038/s41593-024-01848-4

Multimodal transcriptomics reveal neurogenic aging trajectories and age-related regional inflammation in the dentate gyrus

In the format provided by the authors and unedited

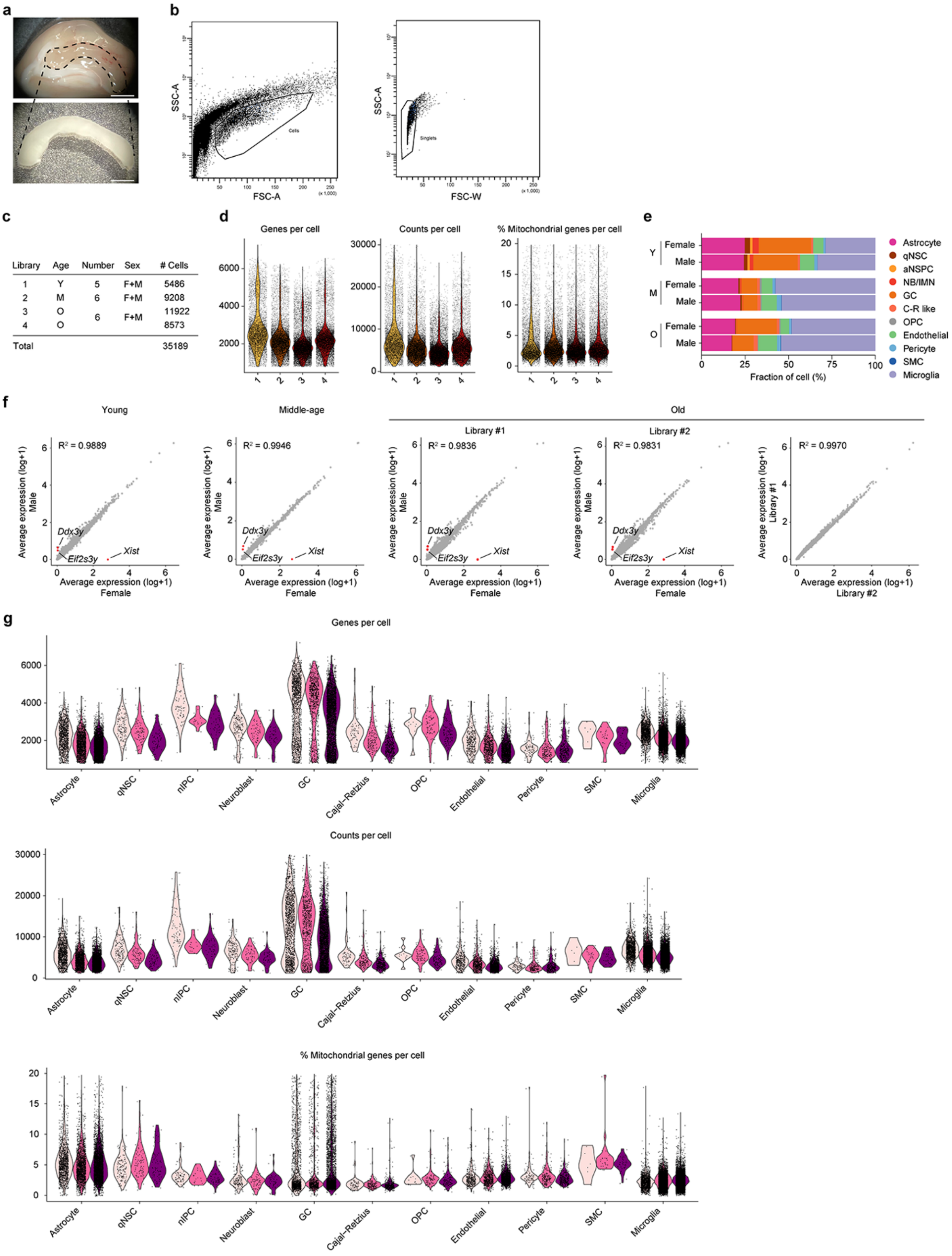

Supplementary Fig. 1 | Characterization and quality control of scRNA-seq dataset.

a, Microdissection of DG. **b**, Gating strategy used to sort single live cells. **c**, Information table of each 10x Chromium library. **d**, Violin plots of the number of gene, count and

the percentage of mitochondrial genes per cells corresponding to different libraries. **e**, Relative proportions of all cell populations in the mouse dentate gyrus corresponding to sex and age. **f**, Correlation between different sexes in each library. For the old age, additional correlation between two libraries using the same animals. **g**, Violin plots of the number of gene, count and the percentage of mitochondrial genes per cells corresponding to each cell type. Scale bars, (**a**, upper) 200 μm and (**a**, lower) 150 μm.

a

Slice	Age	Position	Sex	Replicate	Slice	Age	Position	Sex	Replicate
1	Y	Pos.	M	#1	9	O	Pos.	M	#1
2	Y	Ant.	M	#1, #2	10	O	Ant.	M	#1, #2
3	Y	Pos.	F	#1	11	O	Pos.	F	#1
4	Y	Ant.	F	#1, #2	12	O	Ant.	F	#1, #2
5	M	Pos.	M	#1	13	O	Pos.	M	#1
6	M	Ant.	M	#1, #2	14	O	Ant.	M	#1, #2
7	M	Pos.	F	#1	15	O	Pos.	F	#1
8	M	Ant.	F	#1, #2	16	O	Ant.	F	#1, #2

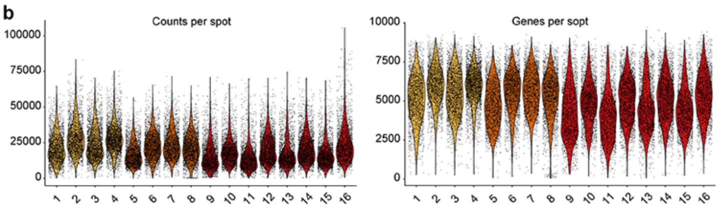

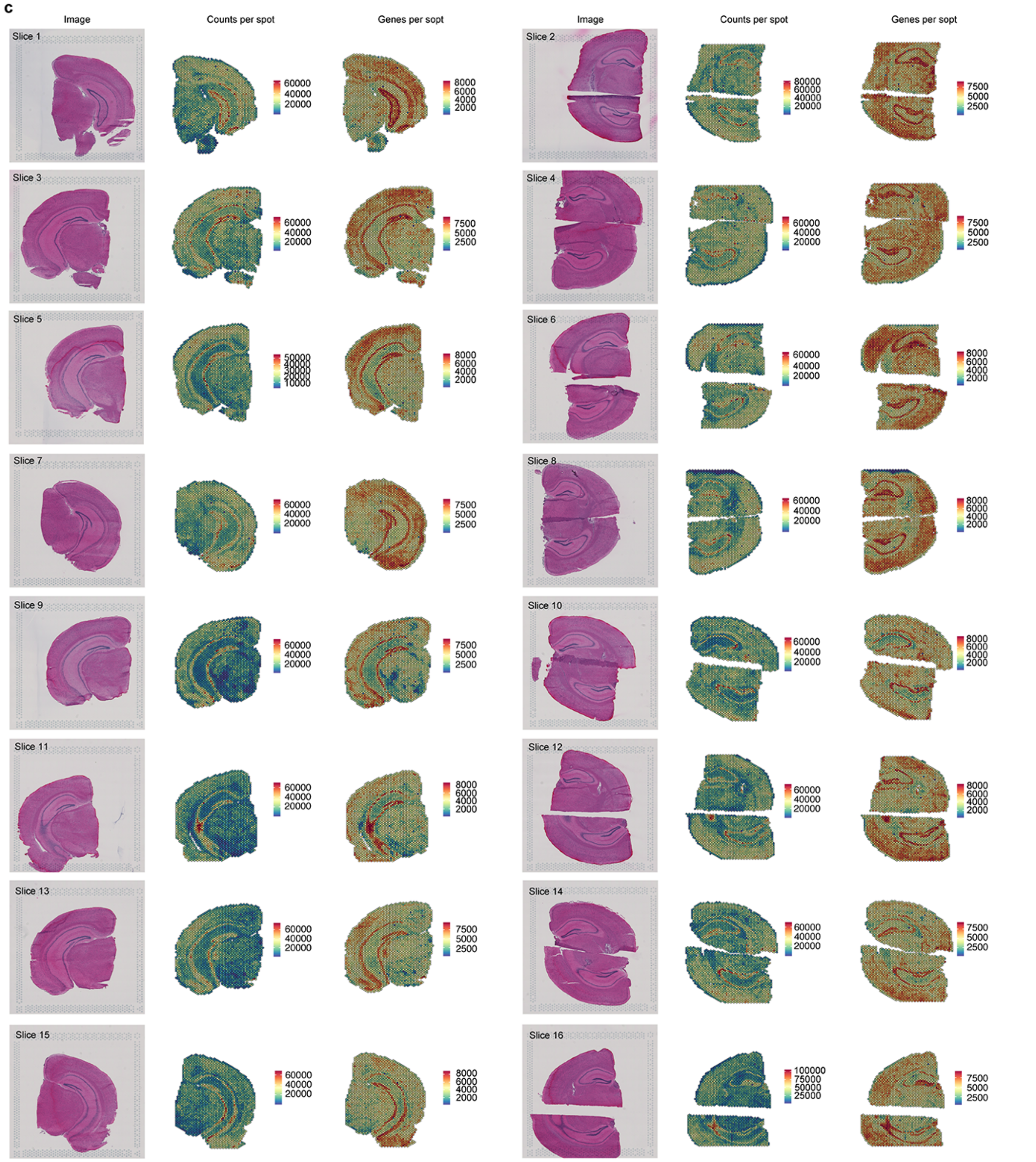

Supplementary Fig. 2 | Characterization and quality control of spatial transcriptomics dataset.

a, Information table of each 10x Visium library. **b**, Violin plots of the number of gene, count and the percentage of mitochondrial genes per cells corresponding to different

libraries. **c**, Feature plots of the number of gene and count on the section of each Visium library. Color gradient indicates the number of counts and genes, respectively.

.
