# Supplementary material for: Multimodal transcriptomics reveal neurogenic aging trajectories and age-related regional inflammation in the dentate gyrus
Source: Nat Neurosci. 2025 Jan 6;28(2):415–30. doi: 10.1038/s41593-024-01848-4 (PMC11802457; doi:10.1038/s41593-024-01848-4)
Supplement: Supplementary file 2 — Reporting Summary [file 41593_2024_1848_MOESM2_ESM.pdf]

nature portfolio

Corresponding author(s): Sebastian Jessberger

Last updated by author(s): Sep 26, 2024

Reporting Summary

Nature Portfolio wishes to improve the reproducibility of the work that we publish. This form provides structure for consistency and transparency in reporting. For further information on Nature Portfolio policies, see our Editorial Policies and the Editorial Policy Checklist.

Statistics

For all statistical analyses, confirm that the following items are present in the figure legend, table legend, main text, or Methods section.

n/a	Confirmed	
☐	☒	The exact sample size (*n*) for each experimental group/condition, given as a discrete number and unit of measurement
☐	☒	A statement on whether measurements were taken from distinct samples or whether the same sample was measured repeatedly
☐	☒	The statistical test(s) used AND whether they are one- or two-sided<br>*Only common tests should be described solely by name; describe more complex techniques in the Methods section.*
☐	☒	A description of all covariates tested
☐	☒	A description of any assumptions or corrections, such as tests of normality and adjustment for multiple comparisons
☐	☒	A full description of the statistical parameters including central tendency (e.g. means) or other basic estimates (e.g. regression coefficient) AND variation (e.g. standard deviation) or associated estimates of uncertainty (e.g. confidence intervals)
☐	☒	For null hypothesis testing, the test statistic (e.g. *F*, *t*, *r*) with confidence intervals, effect sizes, degrees of freedom and *P* value noted<br>*Give P values as exact values whenever suitable.*
☒	☐	For Bayesian analysis, information on the choice of priors and Markov chain Monte Carlo settings
☐	☒	For hierarchical and complex designs, identification of the appropriate level for tests and full reporting of outcomes
☒	☐	Estimates of effect sizes (e.g. Cohen's *d*, Pearson's *r*), indicating how they were calculated

Our web collection on statistics for biologists contains articles on many of the points above.

Software and code

Policy information about availability of computer code

Data collection	No software was used for data collection.
Data analysis	Softwares used: Cell Ranger (v6.0.2, 10x Genomics), Space Ranger (v1.2.0, 10x Genomics), Loupe Browser software (v6, 10x Genomics), ImageJ (v2.9.2), RStudio (v4.0.3), Prism (v9.5.1)<br>R packages used: Seurat (v4.3), DoubletFinder (v2.0.3), STUtility (v0.1.0), msigdbr (v7.4.1), DESeq2 (v1.30.1), harmony (v0.1.0), monocle2 (v2.18.0), Mfuzz (v2.50.0), SCENIC (v1.2.4), GENIE3 (v1.12.0), RcisTarget (v1.10.0), AUCell (1.13.3), caret (v6.0-88), ggplot2 (v3.3.5), ggrepel (v0.9.1), ggridges (v0.5.3), cowplot (v1.1.1), dplyr (v1.0.7), igraph (v1.2.6), alluvial (v0.2-0), MAST (v1.16.0), pheatmap (v1.0.12), RColorBrewer (v1.1-2), viridis (v0.6.1)<br>Python package used: tangram-sc (v.1.0.4)

For manuscripts utilizing custom algorithms or software that are central to the research but not yet described in published literature, software must be made available to editors and reviewers. We strongly encourage code deposition in a community repository (e.g. GitHub). See the Nature Portfolio guidelines for submitting code & software for further information.

Data

Policy information about availability of data

All manuscripts must include a data availability statement. This statement should provide the following information, where applicable:

- Accession codes, unique identifiers, or web links for publicly available datasets
- A description of any restrictions on data availability
- For clinical datasets or third party data, please ensure that the statement adheres to our policy

Data generated and analyzed presented in this study are available under GEO accession number GSE233363.
Processed data (e.g., Seurat object and tables of meta information) for transcriptomics are available at https://github.com/JessbergerLab/AgingNeurogenesis_Transcrptomics.
Public/published database used in this study:
Hochgerner 2018: GSE104323
Dulken 2019: BioProject PRJNA450425
Yao 2021: https://portal.brain-map.org/atlases-and-data/rnaseq
Hahn 2023: GSE212576
AnimalTFDB (v3.0): http://bioinfo.life.hust.edu.cn/AnimalTFDB/#!/

Research involving human participants, their data, or biological material

Policy information about studies with human participants or human data. See also policy information about sex, gender (identity/presentation), and sexual orientation and race, ethnicity and racism.

Reporting on sex and gender	n/a
Reporting on race, ethnicity, or other socially relevant groupings	n/a
Population characteristics	n/a
Recruitment	n/a
Ethics oversight	n/a

Note that full information on the approval of the study protocol must also be provided in the manuscript.

Field-specific reporting

Please select the one below that is the best fit for your research. If you are not sure, read the appropriate sections before making your selection.

☒ Life sciences ☐ Behavioural & social sciences ☐ Ecological, evolutionary & environmental sciences

For a reference copy of the document with all sections, see nature.com/documents/nr-reporting-summary-flat.pdf

Life sciences study design

All studies must disclose on these points even when the disclosure is negative.

Sample size	A total of 17, 12 and 48 mice were used for scRNA-seq, ST and histological experiments in this study, with a total of 35,189 single cell transcriptomes, 42,169 spatial spot transcriptomes and 1048 tissue sections collected. No sample size calculation was performed.
Data exclusions	Cells of poor quality and spots with technical artifacts were excluded from analysis. Detailed description is in the Methods section.
Replication	The main findings in the transcriptomics experiments were validated by histological analysis (immunofluorescent staining and single-molecule RNA FISH). Histological experiments were replicated in at least three biological replicates (for details please refer to methods sections). Replication experiments were successful.
Randomization	No randomization for transcriptomics or histological experiments was possible due to comparison of different age/genotype groups.
Blinding	Transcriptomics experiments were not blinded for group allocation during data acquisition and analysis. Single-molecule RNA FISH and Pdgfbret/ret experimenters were blinded for group allocation during analyses. For the rest histological analysis, due to the nature of aging on neurogenic activity, experimenters are aware of animals from certain ages, such that experimenters were not blinded for group allocation during data acquisition. For the rest histological analysis of microglia and neuroinflammation, experimenters were not blinded for group allocation during analyses.

Reporting for specific materials, systems and methods

We require information from authors about some types of materials, experimental systems and methods used in many studies. Here, indicate whether each material, system or method listed is relevant to your study. If you are not sure if a list item applies to your research, read the appropriate section before selecting a response.

Materials & experimental systems

n/a	Involved in the study
☐	☒ Antibodies
☒	☐ Eukaryotic cell lines
☒	☐ Palaeontology and archaeology
☐	☒ Animals and other organisms
☒	☐ Clinical data
☒	☐ Dual use research of concern
☒	☐ Plants

Methods

n/a	Involved in the study
☒	☐ ChIP-seq
☐	☒ Flow cytometry
☒	☐ MRI-based neuroimaging

Antibodies

Antibodies used

Primary
Antigen Host Source Catalog# Concentration
S100b Rabbit Abcam ab52642 1:500
SOX2 Rat Thermo Fisher Scientific 14-9811-82 1:200
GFAP Chicken Aves GFAP 1:500
Ki67 Rat Thermo Fisher Scientific 14-5698-82 1:500
DCX Guinea pig Millipore ab2253 1:500
NEUROD Goat Santa Cruz sc1084 1:250
IBA-1 Goat Novus NB100-1028 1:500
CD3 Rabbit Novus NB600-1441SS 1:200
CD8a Rat Thermo Fisher Scientific 14-0808-80 1:200
GZMB Goat R&D AF1865 1:100
STAT1 Rabbit Cell Signaling 14994T 1:200
Collagen IV Rabbit Bio-Rad 2150-1470 1:750
CD13 Goat Novus AF2335 1:500
GFP Goat Rockland 600-101-215 1:500
tdTomato Goat Rockland 600-401-379 1:500
tdTomato Goat Sicgen AB8181-200 1:500

Secondary
Antigen Host Source Catalog# Concentration
Alexa Fluor 488 anti-goat IgG (H+L) Donkey Jackson Immuno Research 705-545-147 1:250
Alexa Fluor 488 anti-chicken IgG (H+L) Donkey Jackson Immuno Research 703-545-155 1:250
Alexa Fluor 488 anti-rabbit IgG (H+L) Donkey Jackson Immuno Research 711-545-152 1:250
Alexa Fluor 488 anti-rat IgG (H+L) Donkey Jackson Immuno Research 712-545-153 1:250
Alexa Fluor Cy3 anti-rat IgG (H+L) Donkey Jackson Immuno Research 712-165-153 1:250
Alexa Fluor Cy3 anti-goat IgG (H+L) Donkey Jackson Immuno Research 705-165-147 1:250
Alexa Fluor Cy3 anti-rabbit IgG (H+L) Donkey Jackson Immuno Research 711-165-152 1:250
Alexa Fluor 647 anti-goat IgG (H+L) Donkey Jackson Immuno Research 705-605-147 1:250
Alexa Fluor 647 anti-rabbit IgG (H+L) Donkey Jackson Immuno Research 711-605-152 1:250
Alexa Fluor 647 anti-guinea pig IgG (H+L) Donkey Jackson Immuno Research 706-605-148 1:250

Validation

Validation links and references for primary antibodies:
S100b Rabbit abcam ab52642 https://scicrunch.org/resolver/AB_882426
Sox2 Rat Thermo Fisher Scientific 14-9811-82 https://scicrunch.org/resolver/AB_11219471
GFAP Chicken Aves GFAP https://scicrunch.org/resolver/AB_2313547/mentions?q=&i=rrid:ab_2313547-127:gfap
Ki67 Rat Thermo Fisher Scientific 14-5698-82 https://scicrunch.org/resolver/AB_10854564
DCX Guinea pig Millipore ab2253 https://scicrunch.org/resolver/AB_1586992
NEUROD Goat Santa Cruz sc1084 https://scicrunch.org/resolver/RRID:AB_630922
IBA-1 Goat Novus NB100-1028 https://scicrunch.org/resolver/AB_521594
CD3 Rabbit Novus NB600-1441SS https://www.novusbio.com/products/cd3-antibody-sp7_nb600-1441
CD8a Rat Thermo Fisher Scientific 14-0808-80 1:200 https://scicrunch.org/resolver/RRID:AB_2572860
GZMB Goat R&D AF1865 1:100 https://www.rndsystems.com/products/mouse-granzyme-b-antibody_af1865?gad_source=1&gclid=Cj0KCQjw7ZO0BhDYARIsAFttkCgdLfp5x4DjuTWnNsAb0fkRQp4Qe3iwqc1trYuaEFHGqTIcj5gAFL8aAv88EALw_wcB&gclsrc=aw.ds
STAT1 Rabbit Cell Signaling 14994T https://www.cellsignal.com/products/primary-antibodies/stat1-d1k9y-rabbit-mab/14994?country=CH
Collagen IV Rabbit Bio-Rad 2150-1470 https://scicrunch.org/resolver/AB_2082660
CD13 Goat Novus AF2335 https://scicrunch.org/resolver/RRID:AB_2227288
GFP Goat Rockland 600-101-215 https://scicrunch.org/resolver/AB_218182

tdTomato Goat Rockland 600-401-379 https://scicrunch.org/resolver/AB_2209751
tdTomato Goat Sicgen AB8181-200 https://scicrunch.org/resolver/RRID:AB_2722750

Animals and other research organisms

Policy information about studies involving animals; ARRIVE guidelines recommended for reporting animal research, and Sex and Gender in Research

Laboratory animals	Wild-type mice:<br>C57BL/6JRj; Janvier-labs, France<br>3-month-old, 9-11-month-old and 16-21-month-old mice were used for transcriptomics experiments.<br>3-month-old, 10-month-old, 18-month-old and 24-month-old mice were used for histological experiments.<br>Transgenic mice:<br>Ai14;B6.Cg-Gt(ROSA)26Sortm14 (CAG-tdTomato)Hze; The Jackson Laboratory, 007914<br>(used to breed experimental Gli1-CreERT2::Rosa26-LSL-tdTomato mice)<br>Gli1tm3(cre/ERT2)Alj; The Jackson Laboratory, 007913<br>(used to breed experimental Gli1-CreERT2::Rosa26-LSL-tdTomato mice)<br>Gli1-CreERT2::Rosa26-LSL-tdTomato (e.g., Pilz et al., 2018 Science (doi: 10.1126/science.aao5056))<br>3-month-old, 10-month-old and 17-month-old mice were used.<br>B6.Cg-Tg(Nes-EGFP)1Yamm/Rbrc<br>3-month-old, 10-month-old and 18-month-old mice were used.<br>Pdgfbret/ret (Pdgfb-tm(ret)) (e.g., Lindblom et al., 2003 Genes Dev(doi: 10.1101/gad.266803))<br>6-month-old mice were used.<br>Mice were group housed in ventilated cages (21-23 Celsius, 40-60% humidity) under a 12h dark/light cycle with ad libitum access to food and water.
Wild animals	No wild animals were used in this study.
Reporting on sex	Mice of mixed sex were used.
Field-collected samples	No field-collected samples were used in this study.
Ethics oversight	Animal experiments were approved by the Cantonal Commission for Animal Experimentation of the Canton of Zurich, Switzerland and Stockholms Norra Djurförsöksetiska Nämnd, Sweden in accordance with national and cantonal/county regulations (license numbers ZH190/19; ZH126/20; 20785-2020).

Note that full information on the approval of the study protocol must also be provided in the manuscript.

Flow Cytometry

Plots

Confirm that:

☐ The axis labels state the marker and fluorochrome used (e.g. CD4-FITC).

☒ The axis scales are clearly visible. Include numbers along axes only for bottom left plot of group (a 'group' is an analysis of identical markers).

☒ All plots are contour plots with outliers or pseudocolor plots.

☒ A numerical value for number of cells or percentage (with statistics) is provided.

Methodology

Sample preparation	Mice were first euthanized via cervical dislocation, followed by taking out brains and then microdissection of DG. The dissected DG from the same age were pooled and dissociated using Neural Tissue Dissociation Kit (P) (Miltenyi Biotec) and further cleaned using Myelin Removal Beads II (Miltenyi Biotec) according to manufacturer's instructions. Briefly, pooled tissue was enzymatically digested for 35 minutes at 37°C, followed by manual trituration with fire-polished pipette tips and filtered with 40-µm strainers. Cell suspension was incubated with Myelin Removal Beads for 15 minutes on ice, followed by cleaning with passing through magnet LS column (Miltenyi Biotec). Single cell suspension was sorted with the target number of 50,000 in an influx cell sorter using 130-µm nozzle (BD Influx).
Instrument	BD FACSAria II
Software	Data was collected using BD FACS software (FACSAria II) and analyzed using FlowJo.
Cell population abundance	Samples were sorted at 1000-1500 events/sec using the 130 um nozzle and a cooling unit with sample temperature of 4°C achieving >90% purity by FACS analysis. Cell population abundance in single cells.
Gating strategy	Gating selecting cells and singlets were chosen to enrich single cells.

☒ Tick this box to confirm that a figure exemplifying the gating strategy is provided in the Supplementary Information.
